# The Molecular Chaperone Hsp90 Is Required for Cell Cycle Exit in *Drosophila melanogaster*

**DOI:** 10.1371/journal.pgen.1003835

**Published:** 2013-09-26

**Authors:** Jennifer L. Bandura, Huaqi Jiang, Derek W. Nickerson, Bruce A. Edgar

**Affiliations:** 1 Fred Hutchinson Cancer Research Center, Seattle, Washington, United States of America; 2 German Cancer Research Center (DKFZ) – Center for Molecular Biology Heidelberg (ZMBH) Alliance, Heidelberg, Germany; University of Illinois at Chicago, United States of America

## Abstract

The coordination of cell proliferation and differentiation is crucial for proper development. In particular, robust mechanisms exist to ensure that cells permanently exit the cell cycle upon terminal differentiation, and these include restraining the activities of both the E2F/DP transcription factor and Cyclin/Cdk kinases. However, the full complement of mechanisms necessary to restrain E2F/DP and Cyclin/Cdk activities in differentiating cells are not known. Here, we have performed a genetic screen in *Drosophila melanogaster*, designed to identify genes required for cell cycle exit. This screen utilized a *PCNA-miniwhite^+^* reporter that is highly E2F-responsive and results in a darker red eye color when crossed into genetic backgrounds that delay cell cycle exit. Mutation of *Hsp83*, the *Drosophila* homolog of mammalian Hsp90, results in increased E2F-dependent transcription and ectopic cell proliferation in pupal tissues at a time when neighboring wild-type cells are postmitotic. Further, these *Hsp83* mutant cells have increased Cyclin/Cdk activity and accumulate proteins normally targeted for proteolysis by the anaphase-promoting complex/cyclosome (APC/C), suggesting that APC/C function is inhibited. Indeed, reducing the gene dosage of an inhibitor of Cdh1/Fzr, an activating subunit of the APC/C that is required for timely cell cycle exit, can genetically suppress the *Hsp83* cell cycle exit phenotype. Based on these data, we propose that Cdh1/Fzr is a client protein of Hsp83. Our results reveal that *Hsp83* plays a heretofore unappreciated role in promoting APC/C function during cell cycle exit and suggest a mechanism by which Hsp90 inhibition could promote genomic instability and carcinogenesis.

## Author Summary

Cells must permanently stop dividing when they terminally differentiate for development to occur normally. Maintenance of this postmitotic state is also important, as unscheduled proliferation of differentiated cells can result in cancer. To identify genes important for restraining cell proliferation during terminal differentiation, we performed a genetic screen in *Drosophila* and found that mutation of Hsp90 caused ectopic cell proliferation in differentiating tissues. Hsp90 is a molecular chaperone that is essential for viability in all eukaryotes and has been shown to facilitate the activity of hundreds of “client” proteins. Indeed, several inhibitors of Hsp90 are currently being tested in clinical trials for use as anti-cancer therapeutics due to their ability to silence multiple client oncoproteins simultaneously. Our data suggest that Hsp90 is necessary to halt cell proliferation during differentiation because the protein Cdh1, which is required for normal cell cycle exit, may be a client of Hsp90. As reduced Cdh1 function results in genomic instability and tumorigenesis, our work highlights the need to design more precisely targeted Hsp90 inhibitors for use as cancer treatments.

## Introduction

Proper development depends on the coordination of cell proliferation and differentiation to produce the correct number of cells in space and time. An important component of this is that cells generally exit the cell cycle in G1 and enter a permanently non-proliferative state when they terminally differentiate. In fact, most cells in adult metazoans have exited the cell cycle and lie in this quiescent state. Control of cell cycle exit is also relevant to cancer, as disruption of the postmitotic state can lead to tumorigenesis.

Cell divisions are primarily driven by oscillations in the activity of Cyclin/Cyclin-dependent kinase (Cdk) complexes [1]. S phase entry is promoted by the activity of Cyclin E/Cdk2 kinase. Cyclin A/Cdk1 and Cyclin B/Cdk1 complexes, once activated by Cdc25/Stg phosphatase, then induce the G2/M transition. These oscillations in Cyclin/Cdk activity are themselves controlled by oscillations in cell cycle gene expression and proteolysis. For example, the E2F/DP transcription factor stimulates the expression of many genes important for both S phase and mitosis, including Cyclins, Cdks and Cdc25/Stg phosphatase [2]. Additionally, the Anaphase-Promoting Complex/Cyclosome (APC/C), which is an E3 ubiquitin ligase, triggers the destruction of a plethora of proteins during mitosis, including Cyclins A and B, to initiate mitotic exit and re-entry into G1 [3].

The prevailing model for how cell divisions are halted upon terminal differentiation has invoked both inhibition of G1 Cyclin/Cdk activity by Cyclin-dependent kinase inhibitors (CKIs) and the repression of E2F activity. In several model systems, including humans, mice and flies, E2F/DP functions as either a transcriptional activator or repressor depending on which E2F family member is present in the dimer [4]. As a result, E2F activity can be inhibited by trading repressive E2F family members for activating ones in the E2F/DP dimers, or by association with Retinoblastoma (Rb) protein or Rb-related pocket proteins, which convert activating complexes into repressive ones.

Mammals possess eight E2F genes, three DP family members, three pocket proteins (Rb, p107 and p130), three Cip/Kip type CKIs (p21, p27 and p57) and four INK type CKIs (p15, p16, p18 and p19). The machinery controlling cell cycle progression in *Drosophila melanogaster* is highly conserved with that in vertebrates. However, convenient for studying the roles of these factors in cell cycle exit, flies possess a simpler system with fewer paralogs of each regulatory factor: one activator E2F (E2F1), one repressive E2F (E2F2), one DP, two Rb family proteins (Rbf and Rbf2) and only a single Cip/Kip family CKI (*dacapo*, *dap*) and no INK family CKI [4], [5].

Studies in mammalian model systems indicate that restraining both Cyclin/Cdks and E2F activity constitute important elements of the cell cycle exit mechanism. For example, overexpression of E2F1 or E2F3, both of which are activating E2Fs, in quiescent mammalian cells can induce DNA replication [6]. Consistent with this, cells lacking all three mammalian pocket proteins fail to undergo senescence or arrest in response to serum starvation or contact inhibition [7], [8]. Furthermore, mice mutant for pRB, the Rb-related pocket proteins p107 or p130, or CKIs experience ectopic proliferation in many tissues [9]–[11].

CKIs and Rb family members are also important for cell cycle exit in *Drosophila*. In the fly embryonic ectoderm, ectopic expression of E2F1 can induce an extra cell cycle [12]. Maintenance of cell cycle arrest in the embryonic epidermis requires the repressive activity of *Rbf*, because cells in *Rbf* mutants exit the cell cycle on time but some cells undergo an ectopic S phase later [13]. Importantly, restraining both Cyclin/Cdk and E2F activity was also found to be required for timely cell cycle exit in terminally differentiating neural and epithelial cells of the eye and wing [14], [15]. In fact, ectopic expression of both G1 Cyclin/Cdk and E2F could completely prevent cell cycle exit in both tissues [15].

In addition, the APC/C has been shown to play an important role in cell cycle exit. In the *Drosophila shattered* mutant, which lacks a core APC/C component, cell cycle exit of differentiating photoreceptor cells of the larval eye is delayed [16]. Similarly, deletion of APC/C subunits in mice and fish results in ectopic cell proliferation in terminally differentiated tissues [17], [18]. The APC/C complex is activated by either of two proteins: Cdc20/Fizzy (Fzy), which activates the complex during mitosis, and Cdh1/Fizzy-related (Fzr), which activates the APC/C from the end of mitosis through G1 phase [19]. It is specifically the G1 APC/C^Cdh1^ that is required for normal exit to occur. Loss or inhibition of Fzr in *Drosophila* delays cell cycle exit in the embryonic ectoderm, larval eye, and terminally differentiating pupal eye and wing cells [20]–[24]. Consistent with this, knockout of Cdh1 causes cultured mammalian cells to have difficulty arresting in G1 and results in ectopic proliferation in mice *in vivo* [25], [26].

Despite the proven role these cell cycle regulators play in establishing the postmitotic state, it is clear that additional unknown mechanisms exist that also enforce cell cycle exit upon terminal differentiation. The over-proliferation observed in mice lacking either pocket proteins or CKIs is tissue-specific, indicating that other factors must enforce cell cycle exit in the unaffected tissues [9]–[11]. In flies, mutation of *dap* alone has no effect cell cycle exit in differentiating pupal eye and wing cells [14], [15] and repressive Rbf/E2F/DP function is not required for permanent cell cycle exit *in vivo* [27]–[29]. Furthermore, although mouse fibroblasts lacking all three pocket proteins have difficulty arresting in G1, embryonic stem cells carrying the same mutations can exit the cell cycle when induced to differentiate [30]. In addition, it is unclear how cell cycle exit is coordinated with terminal cell differentiation, and how the cell signaling pathways that promote differentiation interface with the core cell cycle machinery.

Here, we have performed a loss-of-function genetic screen in *Drosophila* to identify mutants defective in cell cycle exit. We demonstrate that *Hsp83* is required for timely cell cycle exit in terminally differentiating pupal eyes and wings. *Hsp83* is the sole *Drosophila* member of the Hsp90 family of molecular chaperones. Generally thought to be less promiscuous than other chaperones, Hsp90 acts to potentiate the functioning of more than 200 client proteins by promoting subtle changes in the structure of those clients [31], [32]. Hsp90 is essential in eukaryotes, and known clientele of Hsp90 include several key signaling pathway proteins, transcription factors and cell cycle proteins. The activity of Hsp90 depends on ATP binding and hydrolysis, which is coupled to conformational changes in Hsp90 protein. In addition, association with co-chaperones and post-translational modifications of Hsp90 protein can modulate its activity.

Analysis of *Hsp83* mutant clones indicates that the cell cycle exit delay is primarily due to increased Cyclin/Cdk activity. In addition, the levels of several APC/C target proteins, including Cyclin A, are increased in cells lacking *Hsp83*. We found that removing one copy of an inhibitor of the APC/C^Cdh1^ could suppress the *Hsp83* cell cycle exit phenotype. These data are consistent with a model in which Cdh1/Fzr is a client protein of Hsp83, and Hsp83 restrains Cyclin/Cdk activity after cell cycle exit by ensuring maximal activity of the APC/C^Cdh1^. In this way, Hsp83 could provide yet another layer of control over the core cell cycle machinery to ensure that cells stop dividing on schedule during development.

## Results

### Identification of a mutant line, *6-55*, that experiences ectopic E2F activity and delayed cell cycle exit

**Figure 1:**
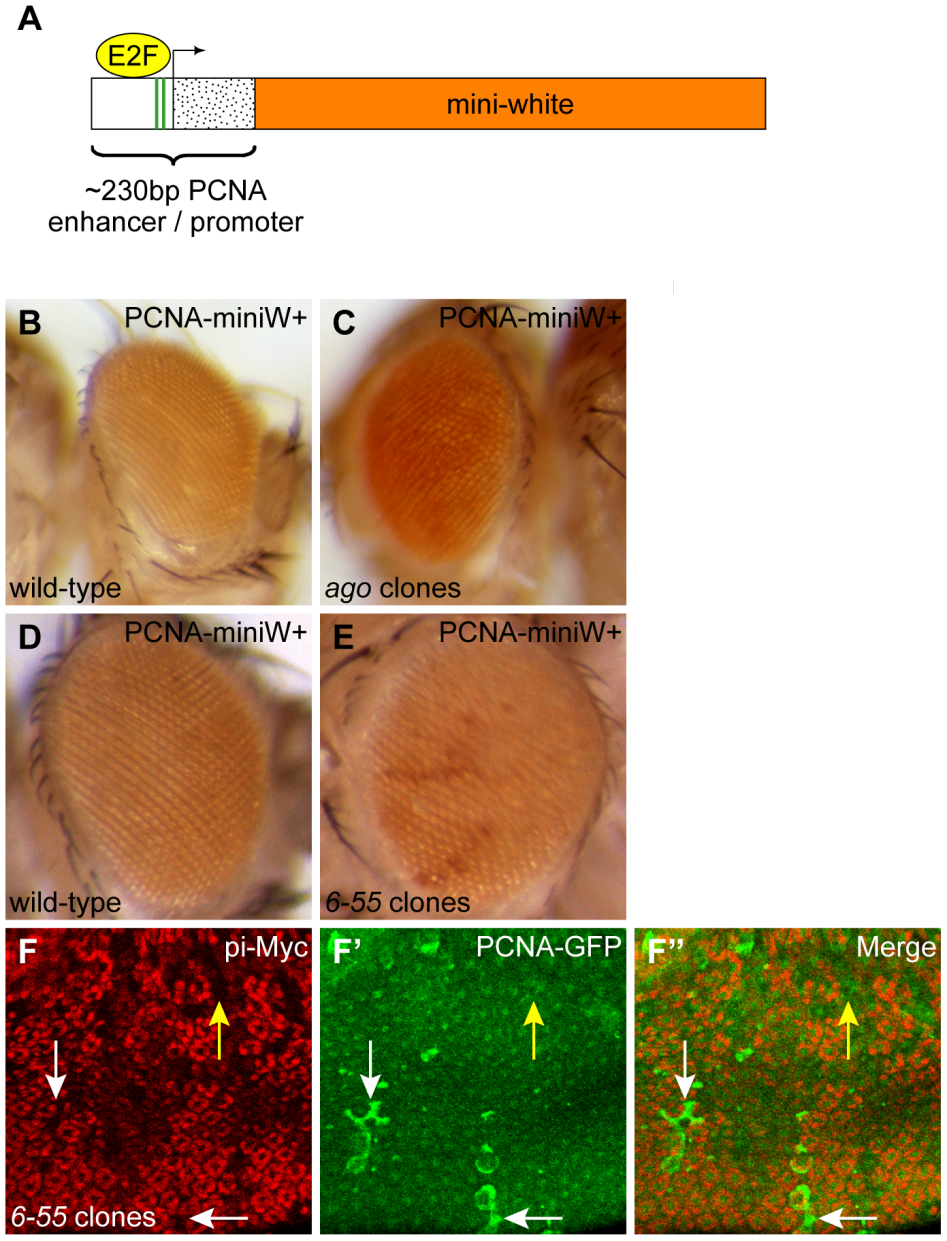
*6-55* mutant cells experience ectopic E2F activity. (A) The *PCNA-miniwhite+* reporter used for screening. This reporter consists of the ∼230 bp enhancer/promoter region from the *PCNA* gene (enhancer, white box; promoter, stippled box), which drives expression of the *mini-white* gene (orange box). The *PCNA* enhancer/promoter contains two E2F binding sites (green stripes) and is highly responsive to E2F activity. (B, C) The *PCNA-miniwhite^+^* phenotype in wild-type adult eyes (B) or eyes from an age-matched individual containing large homozygous clones mutant for *ago* (C). (D, E) *PCNA-miniwhite^+^* in wild-type (D) or an age-matched animal containing homozygous *6-55* mutant clones (E). The *PCNA-miniwhite^+^* transgene conferred a light orange background color in all eyes and a darker red color in the presence of ectopic E2F activity. (F–F″) Expression of the *PCNA-GFP* reporter (green) in *6-55* mutant clones clones marked by the absence of pi-Myc marker (red) in pupal eyes after 24 hour APF. The *PCNA-GFP* reporter was present at low levels in the surrounding non-clone control cells, but some *6-55* mutant cells experienced ectopic *PCNA-GFP* expression at this time (white arrows). The yellow arrow indicates an example of a *6-55* mutant clone that did not display ectopic *PCNA-GFP* expression.

To identify unknown factors required for timely cell cycle exit, we performed a loss-of-function screen utilizing a *PCNA-miniwhite^+^* reporter gene (Figure 1A). Briefly, this reporter consists of a portion of the *proliferating cell nuclear antigen* (*pcna*) promoter/enhancer sequence that is E2F-responsive, and is active in proliferating cells but inactive in non-dividing cells ([15], [33] and data not shown). The *pcna* promoter/enhancer sequence induces expression of the *mini-white* gene, which is a truncated form of the *white* (*w*) gene necessary for red eye color. *white* must be expressed specifically between 1 and 2 days after puparium formation in order for the flies to exhibit red eye color [34]. Just prior to this phenocritical period for *white*, all cells in wild-type pupal eyes permanently exit the cell cycle and exhibit reduced levels of E2F activity by 24 hours after puparium formation (hr APF) [15], [35]. Therefore, animals carrying the *PCNA-miniwhite^+^* reporter only display dark red eye tissue if either ectopic E2F activity or ectopic cell proliferation continues after wild-type cells have become quiescent. We tested the use of *PCNA-miniwhite^+^* to identify mutants that delay cell cycle exit by inducing mutant eye clones of *archipelago* (*ago*) in animals carrying the *PCNA-miniwhite^+^* reporter. *ago*, which is the *Drosophila* homolog of mammalian Fbw7, encodes an F-box protein important for SCF-dependent degradation of Cyclin E and was previously shown to be required for timely cell cycle exit in the *Drosophila* pupal eye [36], [37]. Compared to controls, flies with mutant *ago* eye clones displayed higher levels of *PCNA-miniwhite^+^* expression (Figure 1B, C).

The screen was performed using ethyl methanesulfonate (EMS) as a mutagen, and *eyeless* (*ey*)*-FLP* was used to induce homozygous mutant tissue in the eyes only of flies heterozygous for mutations created on FRT chromosomes. After screening ∼185,500 such animals, we identified and established 61 mutant lines (representing a maximum of 57 genetic complementation groups) that resulted in an increase in *PCNA-miniwhite^+^* reporter gene expression in adult eyes. (For a detailed summary of this screen, refer to Table S1.) Three of the mutant lines (one complementation group) failed to genetically complement mutant alleles of *ago*, indicating that these lines were *ago* mutants. This confirmed the efficacy of the loss-of-function screen. In addition, a mutant line generated on chromosome arm 3L and designated as *l(3)CCE2^6-55^* (*6-55*) also experienced increased expression of *PCNA-miniwhite^+^* compared to controls when homozygosed using *ey-FLP* (Figure 1D,E).

In the course of this study, we used multiple different types of mosaic analysis in larval and pupal tissues to analyze the *6-55* mutant line. FLP/FRT mitotic clones were induced to examine homozygous mutant cells within a heterozygous animal. These were induced in the eye using *ey-FLP*, and were marked by the absence of either GFP or *pi-Myc* labeling, in cases where a GFP+ transgene was to be visualized. To generate GFP-marked homozygous clones in which we could simultaneously overexpress transgenes, we used the MARCM system [38]. Finally, to create clones solely for the overexpression of one or more transgenes, we used the flip-out system in which clones marked with GFP were induced using *heat shock* (*hs*)*-FLP*.

To confirm that the observed increase in *PCNA-miniwhite^+^* expression truly represented an increase in E2F-dependent transcription, we assayed *6-55* homozygous mutant clones in the pupal eye for increased expression of another E2F-responsive reporter, *PCNA-GFP*, which contains the same *pcna* enhancer/promoter region as *PCNA-miniwhite^+^* [33]. After 24 hr APF, increased *PCNA-GFP* expression was apparent in some cells within *6-55* mutant clones, compared to the low level of *PCNA-GFP* expression in either wild-type clones or the wild-type cells surrounding *6-55* mutant clones (Figure 1F–F″ and data not shown). This ectopic *PCNA-GFP* expression was also not observed in *6-55* mutant clones that carried a *PCNA-GFP* reporter lacking the E2F binding sites (Figure S1). These data confirm that E2F activity is increased in *6-55* mutant tissue.

**Figure 2:**
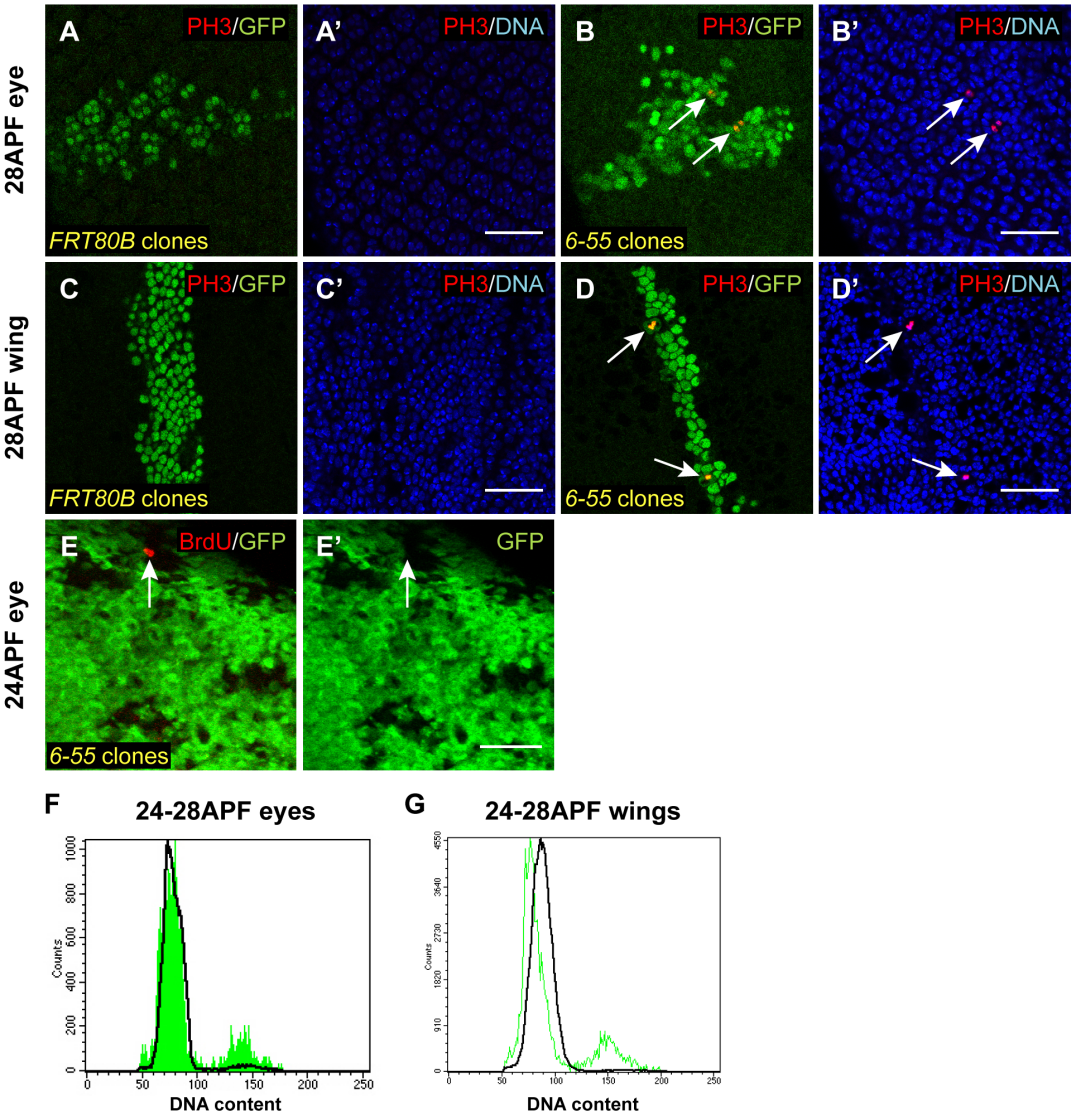
*6-55* mutant cells undergo ectopic cell proliferation. (A–D′) Clones homozygous for a wild-type FRT80B chromosome (A–A′, C–C′) or an FRT80B chromosome carrying the *6-55* mutation (B–B′, D–D′) and also overexpressing GFP and P35, an inhibitor of apoptosis, were induced using the MARCM system. Pupal tissues were then isolated at 28 hr APF and labeled with anti-PH3 antibodies (red) to visualize mitosis. The clones were marked with GFP (green) and DNA was stained with either Hoechst or DAPI (blue). White arrows indicate mitotic *6-55* mutant cells. (E–E′) 24 hr APF FLP/FRT homozygous *6-55* pupal eye clones marked by the absence of GFP (green) were labeled with BrdU (red) to assay for DNA replication. The white arrow in E and E′ indicates a *6-55* mutant cell that has incorporated BrdU. (F, G) Flow cytometry was also used to assess the cell cycle profile of 24–28 hour APF eyes (F) and wings (G) containing *6-55* homozygous MARCM clones marked with GFP and overexpressing P35. GFP-marked clone cells are represented by the green traces, while control non-clone cells are represented by the black traces. At this time point, almost 100% of wild-type cells in both eyes and wings have a G1 (2N) DNA content, whereas approximately 10% of *6-55* clone cells exhibit a G2 (4N) DNA content. All scale bars are 25 µm.

We then wanted to determine whether these *6-55* mutant cells also underwent ectopic cell proliferation. To test this, we labeled pupal eyes and wings from animals containing *6-55* mutant MARCM clones with anti-phospho-Ser10 histone H3 (PH3) antibodies as a marker for mitosis [39]. At 28 hr APF, we never observed PH3 labeling in wild-type clones (Figure 2A–A′ and C–C′). In contrast, a small number of *6-55* mutant cells at this time point labeled with PH3 antibodies (1% in pupal wings) (Figure 2B–B′ and D–D′). In addition, homozygous FLP/FRT clones mutant for *6-55* exhibited BrdU incorporation, a marker for DNA replication, at 24 hr APF when the surrounding control cells were quiescent and did not incorporate BrdU (Figure 2E–E′). We observed evidence of this ectopic cell proliferation until approximately 40 hr APF, but not at time points thereafter (Figure S2).

As an additional assay for inappropriate cell proliferation in *6-55* mutant cells, we used flow cytometry. 24–28 hr APF pupal eyes and wings containing either wild-type or *6-55* GFP-expressing MARCM clones were isolated and the cells were dissociated and stained with the DNA dye Hoechst 33342. Flow cytometric analysis revealed that both the GFP-expressing wild-type control MARCM clone cells and the non-GFP-expressing non-clone cells from all samples possessed a G1 DNA content, indicative of proper cell cycle exit (Figure 2F–G and data not shown). However, the *6-55* MARCM clones contained a small proportion of cells (∼10%) with a G2 DNA content (Figure 2F–G). Combined, these data indicated that at least some *6-55* mutant cells experience a delay in cell cycle exit upon terminal cell differentiation.

### A mutation in *Hsp83* is responsible for the cell cycle exit phenotype in *6-55*

**Figure 3:**
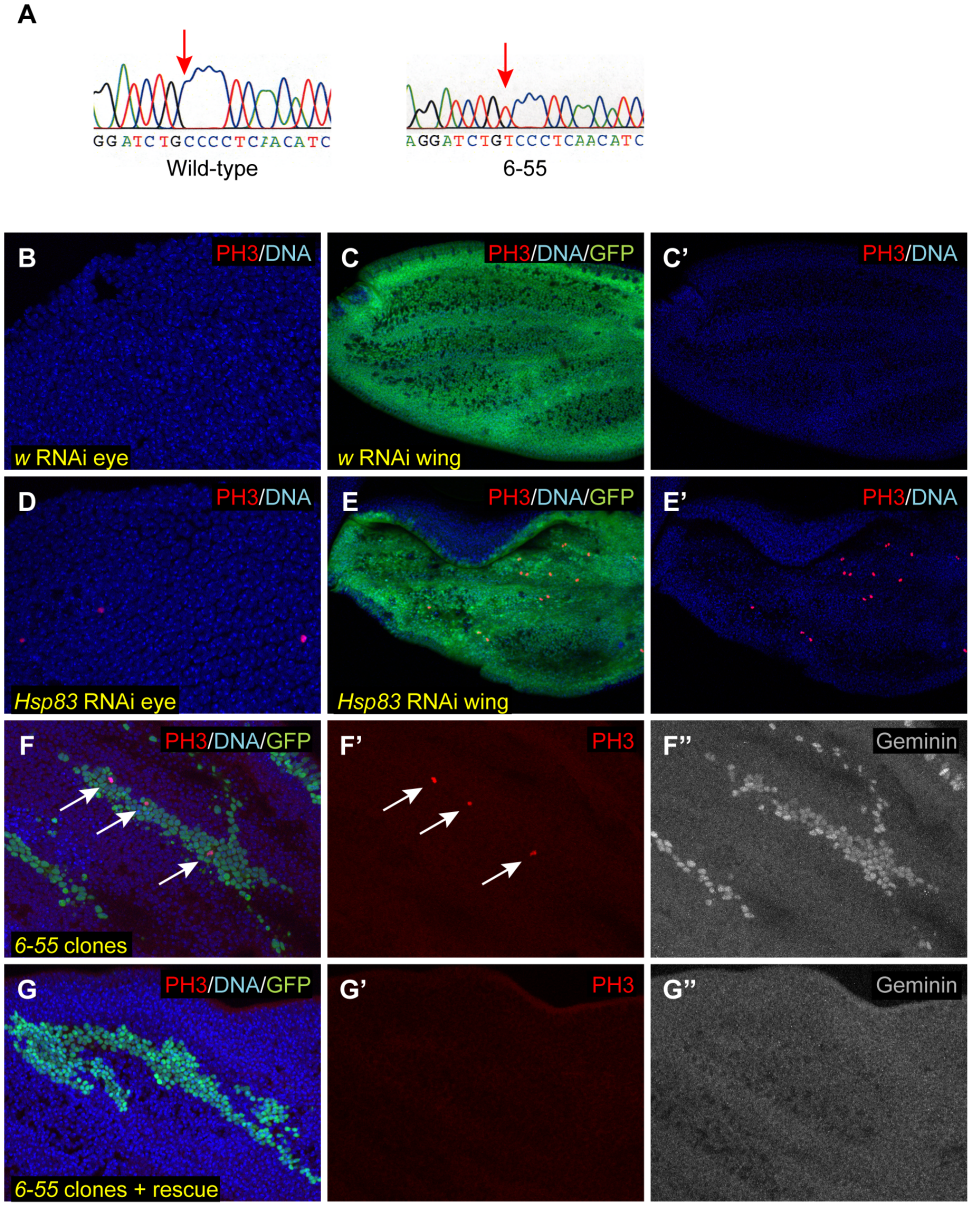
*6-55* is a mutant allele of *Hsp83*. (A) A portion of the ABI sequencing traces from the isogenized parental FRT80B strain and *6-55* homozygous mutant animals. Red arrows indicate the C to T base pair change at 3L coordinate 3195636, which is within the *Hsp83* protein coding region. (B–E′) RNAi directed against *w* (B–C′) or *Hsp83* (D–E′) was expressed in either 28 APF pupal eyes (B, D) or the dorsal half of 26 APF pupal wings (C–C′, E–E′). Pupal tissues were then stained with PH3 (red) to assay for ectopic mitoses and Hoechst to visualize DNA (blue). GFP (green) was also expressed in the dorsal half of the wing (C, E). (F–G″) Homozygous *6-55* MARCM clones overexpressing GFP (green) and P35 were induced in 24–30 APF pupal wings. The animals in G–G″ also carry one copy of the genomic rescue construct for *Hsp83*. These wings were labeled with Hoechst (blue) (F, G), anti-PH3 (red) (F–F′, G–G′), and antibodies against Geminin protein (gray) (F″, G″). White arrows in F and F′ indicate mitotic cells.

Although clones of *6-55* homozygous mutant cells could be produced, animals composed entirely of *6-55* homozygous mutant cells died as pupae (data not shown). We exploited this lethal phenotype for the initial genetic mapping of the molecular lesion responsible for the *6-55* mutant phenotypes. A combination of deficiency and recombination mapping restricted the region of interest in which the gene corresponding to *6-55* resided to an approximately 50 kb region (see Materials and Methods for details). Approximately 40 kb of those 50 kb were sequenced in both the *6-55* mutant strain and the isogenic parental strain mutagenized to create *6-55*. This analysis revealed only one base pair change in *6-55* within those 40 kb: a C to T change at 3L coordinate 3195636, within the *Hsp83* protein coding region, which would be predicted to result in a proline (P) to serine (S) change at residue 380 in the Hsp83 protein (Figure 3A).

*Hsp83* is the *Drosophila* homolog of mammalian Hsp90, a molecular chaperone with an established role in optimizing the function of over 200 known client proteins [32], [40]. Although its expression is induced after cellular stress, Hsp90 also plays an important role in facilitating the activation and/or maturation of its client proteins under non-stress conditions. The proline residue mutated in *6-55* is conserved in the Hsp90 family proteins in other organisms, from yeast to humans [40]. Further, this proline lies within the catalytic loop, a short stretch of 21 amino acids shown to be essential for viability and ATP hydrolysis in yeast Hsp90 [31], [41]. Therefore, it is likely that the *6-55* mutation is a loss-of-function mutation for *Hsp83* and that a similar mutation in the corresponding residue in other organisms would also result in loss-of-function phenotypes.

To confirm that *Hsp83* is indeed non-functional in *6-55*, we performed genetic complementation tests against known mutant alleles of *Hsp83*. *6-55* failed to genetically complement nine alleles of *Hsp83* (Table 1). The *6-55* mutation in trans to two of these alleles (*Hsp83^j5C2^* and *Hsp83^P582^*) was lethal, and was semi-lethal in trans to two other alleles that carry identical molecular lesions (*Hsp83^19F2^* and *Hsp83^13F3^*). In addition, five other *Hsp83* mutant alleles (*Hsp83^e6A^*, *Hsp83^e6D^*, *Hsp83^e1D^*, *Hsp83^e3A^* and *Hsp83^e4A^*) produced viable but male-sterile flies when combined with *6-55*. These phenotypes are all known *Hsp83* mutant phenotypes, and are consistent with the genetic complementation that has observed previously with multiple *Hsp83* alleles [42]. Further, two pieces of evidence led us to believe that the mutation in *6-55* is a hypomorphic mutation. First, the stronger alleles of *Hsp83* are lethal in trans to other *Hsp83* alleles, whereas weaker alleles are often male-sterile in trans to each other [42]. In addition, null alleles of *Hsp83* have been reported to be embryonic lethal; however, *6-55* is pupal lethal [42], [43]. Therefore, the *6-55* allele behaves genetically like a partial loss-of-function mutation in *Hsp83*.

**Table 1:** The *6-55* mutation fails to genetically complement mutant alleles of *Hsp83*.

*Hsp83* allele	Nature of the mutation	Phenotype in trans to *6-55*
j5C2	P element insertion	Lethal
P582	P element insertion	Lethal
19F2	Missense (R48C)	Semilethal
13F3	Missense (R48C)	Semilethal
e6A	Missense (S592F)	Male sterile
e6D	Missense (E317K)	Male sterile
e1D	Missense (S38L)	Male sterile
e3A	Misssense (S574C)	Male sterile
e4A	Missense (S655F)	Male sterile

Although these data indicated that *6-55* contains a mutation in *Hsp83* that is responsible for the lethal phenotype, it was still formally possible that the lesion in *Hsp83* was not responsible for the cell cycle exit phenotype we observed in *6-55* mutant clones. To determine if *Hsp83* is required for timely cell cycle exit, we generated flies that carried both the *6-55* mutation and a genomic rescue construct that contains the entire coding sequence of *Hsp83* [44]. The presence of this genomic rescue construct rescued both the lethality of the *6-55* homozygotes (data not shown) and the cell cycle exit phenotype, as we never observed any PH3 positive cells after 24 hr APF in flies with *6-55* MARCM clones that also carried the rescue transgene (compare Figure 3F–F′ with G–G′). It should be noted that the *Hsp83* genomic rescue construct also contains the entire coding sequence for the neighboring genes *CG14966* and *CG32276* [44]. Although we have not sequenced these genes in the mutant strain to confirm that they are unaltered, both reside in a region that remains intact in the deficiency strain *Df(3L)BSC23* that failed to complement *6-55*. As a consequence, it is unlikely that either of these genes could correspond to *6-55*.

To confirm that *Hsp83* is required for cell cycle exit, we expressed RNAi directed against *Hsp83* either in postmitotic cells in the eye using *GMR-GAL4* or in the dorsal half of the wing using *apterous* (*ap*)-*GAL4* and assayed for ectopic cell proliferation with anti-PH3 antibodies in 28 hr APF pupal tissues. At this time point, we observed multiple mitotic cells in both eyes and wings, which were not seen in controls in which an RNAi against the *white* gene was expressed, or in the ventral half of the wing (Figure 3B–E′ and data not shown). Together, these multiple pieces of data indicated that a loss-of-function hypomorphic mutation in *Hsp83* is responsible for the delay in cell cycle exit seen in the *6-55* mutant cells.

### The cell cycle defect caused by mutation of *Hsp83* is more severe after cell cycle exit than in proliferating tissues

**Figure 4:**
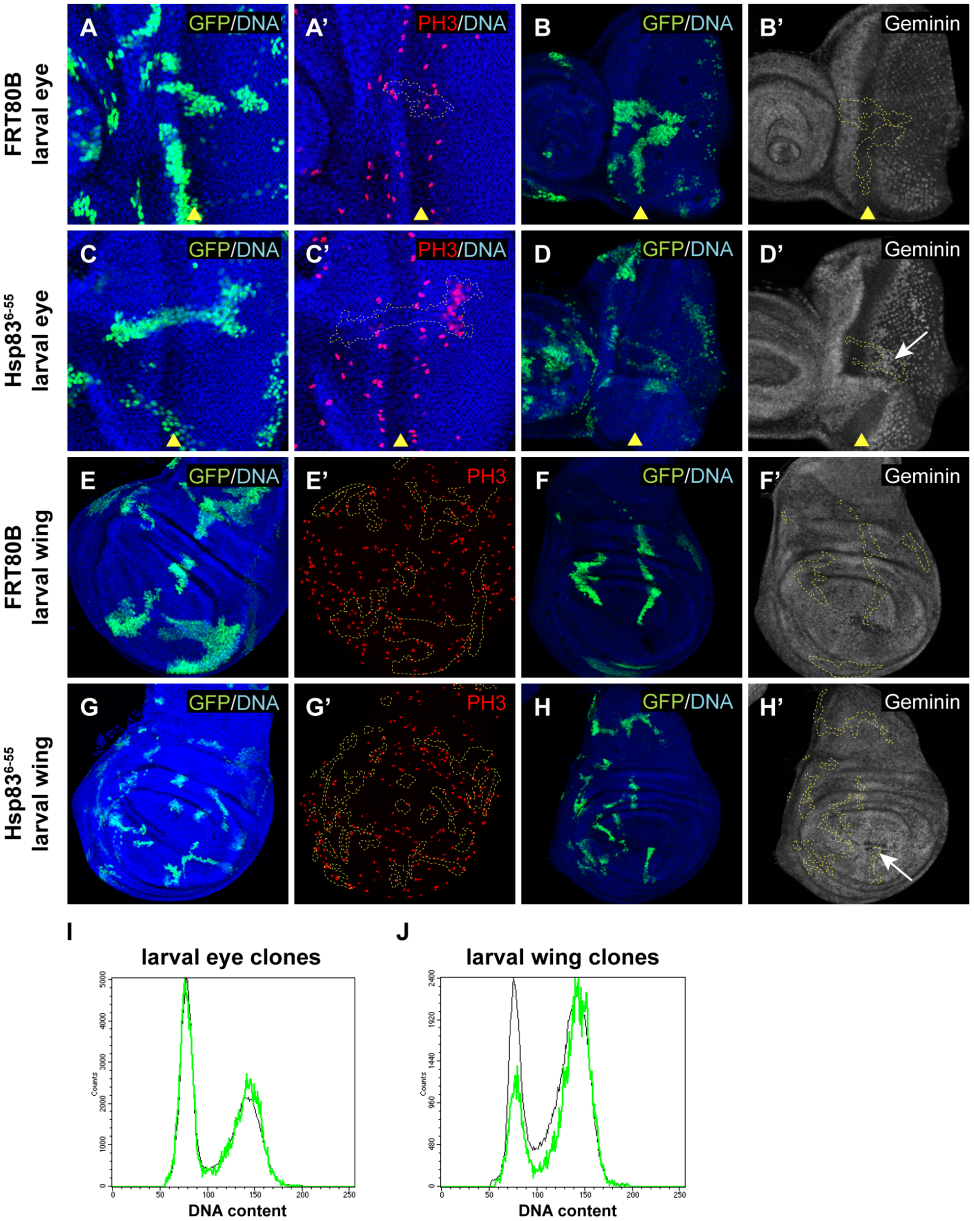
Mutation of *Hsp83* only causes mild cell cycle effects in proliferating cells. (A–H′) Clones homozygous for a wild-type FRT80B chromosome (A–A′, B–B′, E–E′, F–F′) or an FRT80B chromosome carrying the *6-55* mutation (C–C′, D–D′, G–G′, H–H′) and also overexpressing GFP and P35 were induced using the MARCM system. Imaginal discs were isolated from wandering third instar larvae and labeled with anti-PH3 antibodies (red) to label mitosis (A′, C′, E′, G′) or anti-Geminin (white) (B′, D′, F′, H′). In all cases, the clones were marked with GFP (green) and DNA was stained with Hoechst (blue). White arrows in D′ and H′ indicate increased Geminin protein levels within *6-55* clones in regions of the disc containing non-proliferating cells. (I, J) Flow cytometry was also used to assess the cell cycle profile of *6-55* homozygous MARCM clones marked with GFP and overexpressing P35 in larval imaginal eye discs (I) and wing discs (J). GFP-marked clone cells are represented by the green traces, while control non-clone cells are represented by the black traces. The cell cycle profile of *6-55* mutant cells in eye discs was indistinguishable from that of controls, but fewer *6-55* mutant wing disc cells had a G1 (2N) DNA content than control cells. Disc images in A–A′, C–C′, E–E′, G–G′ are maximum projections and in B–B′, D–D′, F–F′, H–H′ are single confocal sections. Anterior is to the right. Yellow arrowheads mark the position of the morphogenetic furrow (MF) in A–D′. Dashed yellow lines outline a single clone spanning the MF in A′, B′, C′, D′ and outline all clones in E′, F′, G′, H′.

We then wondered whether this cell cycle effect of *Hsp83* loss-of-function was specific to cell cycle exit or whether a reduction in *Hsp83* function could also cause ectopic cell divisions in proliferating tissues. To test this, we induced *6-55* mutant MARCM clones in larvae and assayed these clones for cell cycle defects in proliferating 3^rd^ larval instar imaginal discs. Compared to controls, there was no obvious difference in PH3 labeling in *6-55* mutant clones in the proliferating regions of eye and wing imaginal discs (Figure 4A–A′, B–B′, C–C′, D–D′). We did occasionally observe ectopic PH3 labeling within *6-55* mutant clones in the non-proliferating regions of the eye disc posterior to the morphogenetic furrow (MF) (Figure 4C–C′). In addition, it is important to note that when mutant clones spanned the MF, there was also no effect on the timing of expression of Elav, a marker of neuronal differentiation (data not shown). This indicated that differentiation occurs in the *6-55* mutant cells with proper timing, and therefore that the ectopic cell proliferation we saw in these tissues was not due to either delayed development or faulty differentiation.

We also used flow cytometry to assess the cell cycle profiles of *6-55* mutant MARCM clones in larval eye and wing discs. In eye discs, *6-55* mutant clones had a cell cycle profile that was indistinguishable from clones made using a wild-type FRT chromosome and the wild-type non-clone cells within the mutant mosaic discs (Figure 4I and data not shown). In wing discs, however, there was a mild change in cell cycle phasing in *6-55* mutant clones compared to controls, such that a larger proportion of the mutant cells had a G2 DNA content and fewer had a G1 DNA content (Figure 4J). This is consistent with either a shortening of G1 phase in these mutant cells or a lengthening of G2. Overall, these data demonstrated that a reduction of *Hsp83* function sufficient to delay cell cycle exit has only a very mild effect in actively proliferating cells.

### The cell cycle exit defect in *Hsp83* mutant cells is caused primarily by an increase in Cyclin/Cdk activity

**Figure 5:**
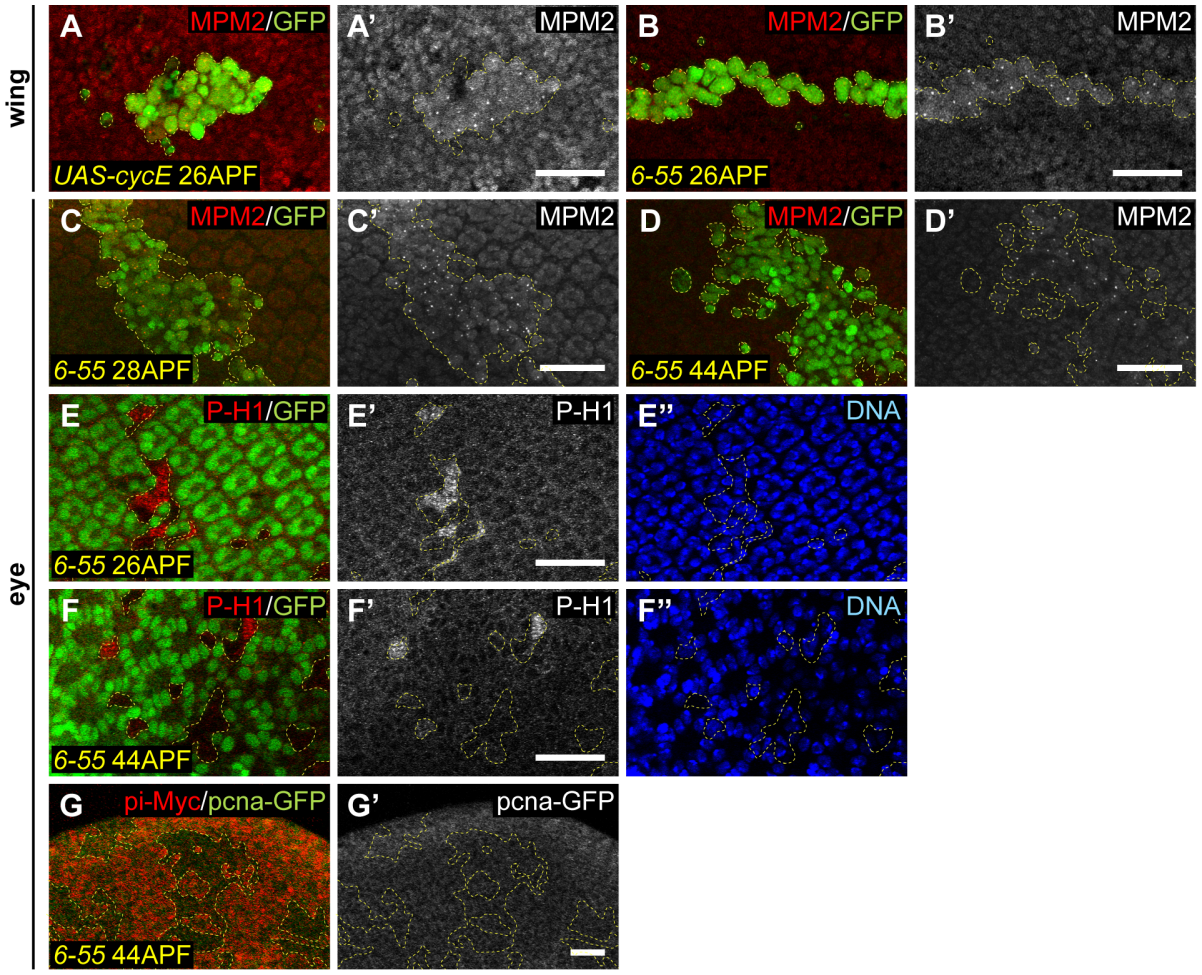
*Hsp83* mutant cells experience ectopic Cyclin/Cdk activity. (A–B′) Labeling of 26 hr APF pupal wings containing MARCM clones (GFP, green) with MPM2 antibody (red in A, B; gray in A′, B′), which is a reporter for Cyclin/Cdk activity. (A, A′) As a control, *cyclin E* overexpression in clones resulted in small foci of MPM2 staining only within clone cells. (B, B′) MPM2 staining of a pupal wing containing a clone of cells mutant for *Hsp83*. Note the presence of foci within the clone but not in the surrounding control cells. (C–F′) Pupal eyes containing *Hsp83* mutant clones were labeled with two markers of Cyclin/Cdk activity: MPM2 (C–D′) and P-H1 (E–F″). (C, D) MARCM mutant clones also overexpressing P35 are marked by the presence of GFP (green). Staining for MPM2 is red in (C, D) and gray in (C′, D′). (E, F) FLP/FRT mutant clones are marked by the absence of GFP (green). Staining for P-H1 is red in (E, F) and gray in (E′, F′). DNA stain is blue (E″, F″). Positive feedback between Cyclin/Cdk activity and E2F activity is still intact in the 26–28 APF eyes in (C–C′, E–E″), but is gone in the 44 APF eyes in (D–D′, F–F″). (G–G′) 44 APF pupal eyes were assayed for a reporter of E2F activity, *PCNA-GFP* (green in G, gray in G′). *Hsp83* mutant FLP/FRT clones are marked by the absence of pi-Myc labeling (red in G). Dashed yellow lines outline the clones. All scale bars are 25 µm.

It had previously been shown that the suppression of both Cyclin/Cdk activity and E2F transcriptional activity are necessary for timely cell cycle exit to occur in terminally differentiating tissues [14], [15], [45]. Clearly, at least some *Hsp83^6-55^* mutant cells possess inappropriate E2F activity after wild-type cells have exited the cell cycle (Figure 1). However, we also wanted to determine if *Hsp83^6-55^* mutants experience ectopic Cyclin/Cdk activity after cell cycle exit. To test this, we utilized two reporters for Cyclin/Cdk activity: anti-phospho-histone H1 antibodies, which detect phospho-epitopes of multiple Cyclin/Cdk complexes in several systems, but which have been shown to specifically respond to Cyclin E/Cdk2 activity in *Drosophila* endoreplicating follicle cells [46], [47], and the MPM2 antibody, which detects a small focus of nuclear staining at the histone locus body (HLB) in response to Cyclin E/Cdk2 activity ([48], [49] and Figure 5A–A′). At both early time points after wild-type cells had exited the cell cycle (26–28 hr APF) and a later time point (44 hr APF), *Hsp83^6-55^* mutant cells exhibited labeling with both of these markers of Cyclin/Cdk activity, whereas the control cells surrounding the *Hsp83^6-55^* mutant clone did not (Figure 5B–F″). This indicated that cells with reduced Hsp83 function retain Cyclin/Cdk activity after wild-type cells have exited the cell cycle.

Given that *Hsp83^6-55^* mutant cells experienced increases in both E2F activity and Cyclin/Cdk activity compared to controls in differentiating tissues, we wondered which of these two effects represented the primary effect of loss of *Hsp83*. At early time points after cell cycle exit in this system (24–36 hr APF), ectopic Cyclin/Cdk activity can still activate E2F activity, and ectopic E2F activity is still capable of stimulating Cyclin/Cdk activity [15]. However, after 36–40 hr APF, crosstalk between Cyclin/Cdk activity and E2F activity is eliminated and neither one can activate the other [15]. Therefore, we determined whether the ectopic E2F activity in *Hsp83^6-55^* mutant clone cells, which had been observed at early times after cell cycle exit, continued after prolonged exit. At 44 hr APF, activation of the *PCNA-GFP* reporter was no longer visible in *Hsp83^6-55^* mutant clones, indicating that ectopic E2F activity was no longer present in *Hsp83^6-55^* cells at this time (Figure 5G–G′). This suggested that increased Cyclin/Cdk activity is the primary defect responsible for the cell cycle exit delay in *Hsp83^6-55^* mutant cells, and that the observed increase in E2F activity was merely a secondary effect caused at early time points after cell cycle exit due to ectopic Cyclin/Cdk activity. Consistent with this interpretation, there were still markers of ectopic Cyclin/Cdk activity at 44 hr APF, a time when *Hsp83^6-55^* mutant cells no longer divided. However, the timing of cell proliferation in *Hsp83^6-55^* mutants (until approximately 40 hr APF) was similar to that seen when either Cyclin E/Cdk2 or Cyclin D/Cdk4 were directly overexpressed in pupal tissues [15].

### *Hsp83^6-55^* mutant cells accumulate proteins targeted for degradation by the APC/C

**Figure 6:**
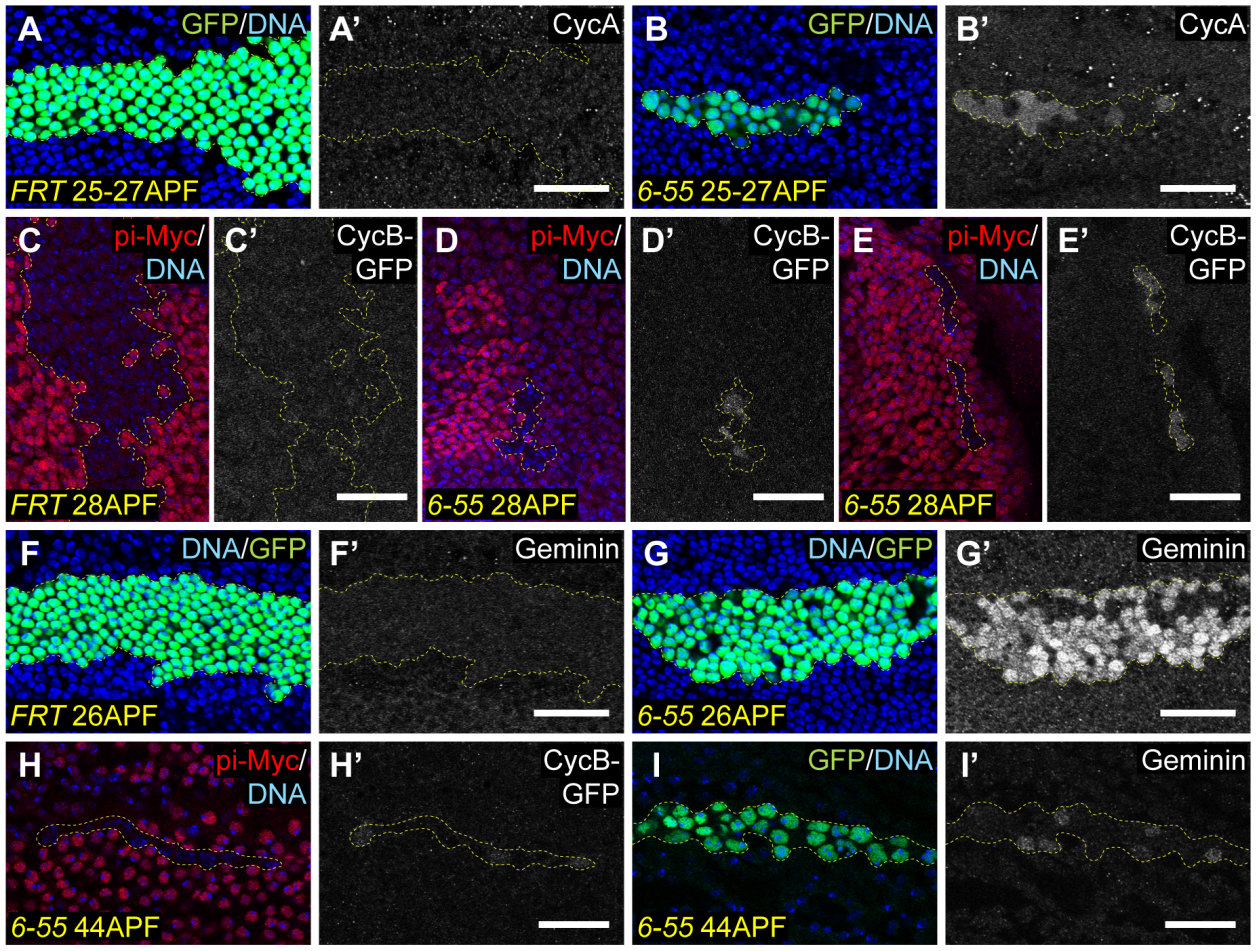
*Hsp83* mutant clones accumulate proteins targeted by the APC/C. *Wild-type* (A–A′, C–C′, F–F′) or *Hsp83* mutant (B–B′, D–E′, G–I′) clones in pupal tissues were assayed for the presence of Cyclin A protein (A–B′), a Cyclin B-GFP fusion protein (C–E′, H–H′) or Geminin protein (F–G′, I–I′) (gray). (A–B′, F–G′, I–I′) Clones were induced using the MARCM system, also overexpress P35, and are marked by the presence of GFP (green); (C–E′, H–H′) clones are FLP/FRT clones and are marked by the absence of the pi-Myc label (red). Dashed yellow lines outline the clones. (A–B′, E–I′) Pupal wings; (C–D′) pupal eyes. (A–G′) show clones in 25–28 APF pupal tissues, shortly after wild-type cells have exited the cell cycle, whereas (H–I′) contain images of clones at 44 APF, when all cell divisions have stopped in *Hsp83* mutant clones. In all cases DNA was stained with either Hoechst or DAPI (blue). All scale bars are 25 µm.

As *Hsp83^6-55^* mutant cells experienced ectopic Cyclin/Cdk activity after they should have exited the cell cycle, we wanted to determine if these cells contained higher Cyclin protein levels than quiescent control pupal eye and wing cells. To test for this, we labeled pupal eyes and wings containing either *Hsp83^6-55^* or wild-type MARCM clones with antibodies against Cyclin A, Cyclin D, and Cyclin E. In addition, we looked at expression of a Cyclin B-GFP fusion protein driven by the endogenous *Cyclin B* promoter (CycB-GFP) in FLP/FRT *Hsp83^6-55^* and control clones. We observed increased levels of both Cyclin A and CycB-GFP in *Hsp83^6-55^* mutant clones compared to wild-type clones and surrounding control cells (Figure 6A–E′). No increase in Cyclin D protein was observed, and increased Cyclin E protein was evident in only some cells of the eye and not at all in the wing blade (data not shown). These data suggested that increases in Cyclin A and/or Cyclin B may be responsible for the ectopic Cyclin/Cdk activity present in *Hsp83^6-55^* mutant tissue.

Cyclin A and Cyclin B are both well-established targets of the Anaphase Promoting Complex/Cyclosome (APC/C) [50]–[52]. The increased protein levels we had observed of both of these led us to hypothesize that APC/C function might be reduced in *Hsp83* mutant cells. Indeed, reducing the function of either a core component of the APC/C or one of its activators, Cdh1/Fzr, has been shown to delay cell cycle exit [16], [20], [21], [23], [24]. To test for a potential APC/C defect, we assayed control and *Hsp83^6-55^* mutant clones for Geminin protein, another known target of the APC/C. As can be seen in Figure 6F–G′, Geminin protein levels were also dramatically increased in *Hsp83* mutant clones compared to control clones or neighboring wild-type cells. Importantly, the observed increase in Geminin levels in *Hsp83^6-55^* mutant clones could be rescued by the *Hsp83* genomic rescue construct (Figure 3F″ and G″). We also assayed Geminin levels in third instar larval discs (Figure 4B–B′, D–D′, F–F′, H–H′). Consistent with our previous experiments in larval discs, there was no apparent difference in Geminin levels between mutant and control clones in proliferating cells. However, *Hsp83^6-55^* clones in the differentiating cells posterior to the MF in the eye and in the G1-arrested cells in the posterior half of the zone of non-proliferating cells (ZNC) exhibited increased Geminin [53], [54]. Altogether, these data indicate that proteins targeted by the APC/C for degradation accumulated in *Hsp83* mutant cells.

The presence of increased amounts of Cyclin A, Cyclin B and Geminin in *Hsp83* mutant cells could be due to decreased degradation of these proteins because of hindered APC/C function, but also could be due to increased expression of these proteins. All three are targets of E2F, which was ectopically active in *Hsp83* mutant cells at time points shortly after wild-type cells have stopped dividing (Figure 1D–F″). To test whether Cyclins A and B and Geminin accumulated in *Hsp83* mutant cells due to ectopic E2F activity, we assayed *Hsp83* mutant clones for CycB-GFP and Geminin at 44 hours APF, a time when E2F was no longer active in these clones (Figure 5G–G′). We found that Geminin and, to a lesser extent, CycB-GFP were still present in *Hsp83* clones at this time point (Figure 6H–I′). Based on this, we feel it is unlikely that Cyclin A, Cyclin B and Geminin accumulated in *Hsp83* mutant cells due to ectopic E2F-dependent transcription.

An additional situation that could lead to accumulation of APC/C target proteins for reasons other than reduced APC/C function is if the *Hsp83* mutant cells were arrested in the G2 phase of the cell cycle. Cyclins A and B and Geminin are all abundant in G2 because the APC/C is not active during this cell cycle stage. To determine whether the *Hsp83* mutant cells containing elevated levels of these proteins were simply arrested in G2, we quantified the number of mutant cells ectopically expressing Geminin and found that in 29–30 hr APF wings, 43% of *Hsp83* mutant cells labeled with antibodies against Geminin (data not shown). Conversely, based on our FACS analysis, only ∼10% of cells within *Hsp83* mutant clones in 24–28 APF wings were in G2 (Figure 2G). These data indicate that the majority of *Hsp83* cells containing elevated levels of Geminin were in G1 phase, and therefore that a G2 arrest cannot be the cause of APC/C target protein accumulation in *Hsp83* mutant cells.

Rather, we favor a model wherein the Cdh1/Fzr activator of the APC/C is a client protein of Hsp83. In addition to a known requirement for the APC/C and Cdh1/Fzr in cell cycle exit, Cdh1/Fzr functions specifically during G1 [19]. Therefore, it is the most likely APC/C component to give primarily a cell cycle exit phenotype when its activity is reduced. According to our hypothesis, Hsp83 would normally function to optimize the activity of the APC/C^Cdh1^, which promotes the degradation of mitotic Cyclins and several other known target proteins [51]. However, when the activity of Hsp83 is reduced by mutation or inhibition by RNAi, APC/C^Cdh1^ function would be hindered, allowing target proteins to accumulate during G1. The increased levels of one or several of these proteins could then stimulate an ectopic cell division in cells that should be postmitotic.

To test whether our hypothesis that Cdh1/Fzr is a client protein of Hsp83 is correct, we asked whether reducing the copy number of *Regulator of cyclin A1* (*Rca1*) could suppress the cell cycle exit phenotype resulting from *Hsp83* loss-of-function. Rca1 is the *Drosophila* homolog of mammalian Emi1. It specifically inhibits the APC/C^Cdh1^ and *Rca1* mutants had previously been shown to dominantly suppress a rough-eye phenotype resulting from elevated CycA/Cdk activity [55], [56]. *hs-FLP*-induced flip-out clones expressing an *Hsp83* RNAi, Dcr-2, P35 and GFP exhibited a mitotic index of 1.24±0.08 in 28 hr APF pupal wings, whereas control clones that only express Dcr-2, P35 and GFP have a mitotic index of 0.00. Interestingly, the mitotic index of *Hsp83* RNAi clones was decreased to 0.65±0.07 when generated in animals heterozygous for the deletion *Df(2L)ED6569*, which removes ∼212 kb of genomic sequence, including the *Rca1* gene. We believe the genetic suppression seen in *Df(2L)ED6569* heterozygotes was due to deletion of *Rca1*, because we also performed this experiment with two mutant alleles of *Rca1*, *Rca1^2^* and *Rca1^1X^*. In an *Rca1^2^* heterozygous background, the mitotic index of *Hsp83* RNAi flip-out clones was also decreased significantly, to 0.81±0.10. However, we did not see suppression of the *Hsp83* RNAi cell cycle exit phenotype in *Rca1^1X^* heterozygotes (mitotic index = 1.31±0.14). The most likely explanation for this discrepancy is that *Rca1^1X^* is probably a weaker allele than *Rca1^2^*. Indeed, neither the nature nor the relative strengths of these alleles has been reported to date. Given the significant effect we observed with both *Rca1^2^* and a deficiency that completely removes *Rca1*, these data support our hypothesis that Cdh1/Fzr is a client protein of Hsp83.

## Discussion

Several mechanisms have been implicated in controlling permanent cell cycle exit upon terminal differentiation. E2F and Rb family members influence cell proliferation by transcriptional regulation, Cyclin/Cdks and CKIs regulate cell proliferation via control of cell cycle protein phosphorylation, and the APC/C targets pro-proliferative proteins for proteasome-dependent degradation. However, it has been clear that additional factors must exist that modulate the activity of known cell cycle regulators to ensure timely cell cycle exit. In this study, we provide evidence of an additional layer of control imposed on the cell cycle machinery by the molecular chaperone Hsp90.

### A role for Hsp90 in cell cycle exit

Cells mutant for the *Drosophila* Hsp90 homologue, *Hsp83*, or expressing an RNAi against *Hsp83* experienced a delay in cell cycle exit. *Hsp83* mutant cells also experienced ectopic increases in both E2F and Cyclin/Cdk activity. Our data indicate that the increased Cyclin/Cdk activity was the primary defect, which secondarily caused an increase in E2F activity only at time points shortly after wild-type cells have exited the cell cycle. We have further found that the *Hsp83* mutant cells contained increased levels of Cyclin A, Cyclin B and Geminin proteins, consistent with reduced APC/C function. This finding has led us to hypothesize that Cdh1/Fzr may be a client protein of Hsp83. The cell cycle exit effect resulting from Hsp83 knock-down was suppressed by reducing the dosage of *Rca1*, which provided support to our hypothesis.

Although the number of cells undergoing mitosis in the mutant and RNAi clones was significantly increased compared to control clones (1–1.24% versus 0%), *Hsp83* loss-of-function resulted in a rather small number of cells delaying cell cycle exit. Why was this? One reason is that the cell cycle exit mechanism is quite robust. To provide a comparison, *rbf1* mutant clones in pupal tissues exhibit a mitotic index of ∼9% only from 24–28 APF and *dap* mutant cells do not experience any cell cycle exit delay [15]. Although the *Hsp83* mutant cells have a mitotic index of only ∼1%, ectopic cell divisions continue until approximately 40 APF, timing consistent with one extra cell division. In addition, direct overexpression of Rca1 to inhibit Fzr delays cell cycle exit with similar timing as the *Hsp83* mutation, and also results in a modest increase in mitotic index in pupal tissues [23]. It is not surprising that *Hsp83^6-55^*, a hypomorphic mutation in something that we believe optimizes the function of Cdh1/Fzr, would produce a much more subtle effect.

To our knowledge, this is the first report providing evidence that Cdh1/Fzr could be an Hsp90 client. In budding yeast, it has been demonstrated that another chaperone, the CCT chaperonin, is required for the folding of both Cdc20 and Cdh1 and therefore is necessary for all APC/C activity [57]. While the CCT chaperonin functions in the bulk folding of nascent proteins, the Hsp90 family of chaperones generally promotes more subtle structural changes to potentiate the function of its clientele. We propose that Hsp90 is specifically required to optimize the function of APC/C^Cdh1^ and not APC/C^Cdc20^. Indeed, the fact that we are able to observe Hsp90 mutant cells undergoing mitosis indicates that the function of the APC/C^Cdc20^ is intact, as cells lacking functional Cdc20/Fzy arrest in mitosis [57]–[59].

Several studies have demonstrated that mutation or inhibition of *fzr* can delay cell cycle exit [20]–[24], but it is not clear which target protein(s) of the APC/C^Cdh1^ are responsible for this delay. One likely candidate for the crucial target protein that causes the delay in cell cycle exit is Cyclin A. In *Drosophila*, Cyclin A normally functions during mitosis, but when ectopically expressed it can also induce entry into S phase [55], [60]. Cyclin A overexpression can drive the G1/S transition even in the absence of Cyclin E, suggesting that Cyclin A/Cdk complexes can directly phosphorylate Cyclin E/Cdk2 targets important for S phase. Further, in mammals it has been demonstrated that Cdk1 is the only essential Cdk and that it is sufficient to drive the entire cell cycle in the absence of interphase Cdks [61]. Indeed, direct overexpression of Cyclin A can cause one complete ectopic cell cycle in differentiating pupal eyes and wings [15]. However, the extra cell cycle induced in the *Drosophila* embryonic epidermis by mutation of *fzr* cannot be rescued by also mutating *cyclin A* [62], suggesting there may be other crucial target proteins of the APC/C^Cdh1^ in addition to Cyclin A that must be restrained in order to initiate cell cycle exit. APC/C targets that could potentially play a role in cell cycle exit include Cyclins B and B3, Cdc25/Stg phosphatase and the DNA replication factor Orc1. Further studies are needed to address whether restraining these target proteins comprises an important part of the cell cycle exit requirement for APC/C^Cdh1^.

### Hsp90: A post-translational mechanism to control the activity of proteins in a cell cycle-dependent manner

Our model is that Hsp90 facilitates the function of the Cdh1/Fzr. What could be the purpose for this regulation of the APC/C^Cdh1^? Unlike other ubiquitin ligases that recognize their targets only when they have been post-translationally modified, for example by phosphorylation or hydroxylation, the APC/C recognizes different unmodified substrates at distinct points in the cell cycle [3]. Although it is not entirely understood how this change in substrate specificity occurs, it is clear that it is partially dependent on which co-activator, Cdc20/Fzy or Cdh1/Fzr, is associated with the complex [3]. While Cdc20/Fzy is expressed periodically and this may partially control activation of the APC/C^Cdc20^ [63], Cdh1/Fzr is not periodically transcribed [19], [63]. Cdh1/Fzr is known to be negatively regulated by both Cyclin/Cdk phosphorylation and binding of inhibitors [3]. In addition, activation of Cdh1/Fzr by interaction with Hsp90 in a cell cycle-dependent manner could potentially also be responsible for the timely activation of the APC/C^Cdh1^.

How could a protein such as Hsp90 that is expressed ubiquitously and at high levels regulate another protein so that it only acts at a specific place and time? Hsp90 function is modulated both by association with co-chaperones and via post-translational modifications, including phosphorylation, acetylation and S-nitrosylation, all of which can direct Hsp90 to particular client proteins [32]. A recent study has even indicated that a portion of cellular Hsp90 is phosphorylated by Wee1, causing it to selectively associate with only some of its clients in a cell cycle-dependent manner [64]. Although it has not been demonstrated that this specific phosphorylation event plays a role during exit from the cell cycle, these data combined with data indicating that Hsp90 is subject to many post-translational modifications [32], [65] raise the possibility that a particular combination of modifications on Hsp90 could create a G1-specific Hsp90 activity that optimizes the function of clients important for cell cycle exit in G1.

### Hsp90 inhibition and cancer

Hsp90 is needed to protect a number of mutated and overexpressed oncoproteins, such as ErbB2/HER2, v-Src, Akt and Bcr-Abl, from misfolding and degradation [32], [66]. Therefore, inhibition of Hsp90 is an efficient way to silence multiple oncogenic signaling pathways simultaneously. As a result, several Hsp90 inhibitors are currently being developed and tested as anti-cancer therapeutics. However, the pleiotropic effects of Hsp90 inhibitors may complicate their clinical efficacy. For example, Hsp90 inhibition results in the release and activation of the heat shock transcription factor 1 (HSF1), and HSF1 has a clear role in supporting the proliferation and survival of transformed cells [67]–[69]. In addition, in *Drosophila*, is had been demonstrated that mutation or inhibition of Hsp90 relieves the suppression of transposon activity, resulting in *de novo* mutations and revealing that Hsp90 inhibition can be mutagenic [70]. Our findings, which suggest that APC/C^Cdh1^ function is reduced in the absence of Hsp90, identify an additional mechanism by which Hsp90 inhibition could promote genomic instability and carcinogenesis. Mouse and human cells lacking Cdh1/Fzr exhibit multiple markers of genomic instability, including chromosome breaks, anaphase bridges and aneuploidy [26], [71]. Further, Cdh1/Fzr heterozygous mice displayed an increased propensity to develop epithelial tumors, suggesting a tumor suppression function for Cdh1/Fzr [26]. Overall, these data highlight a need to target Hsp90 inhibitors not only to tumor cells, but specifically to Hsp90-oncoprotein client interactions within tumor cells. Current efforts by researchers to design Hsp90 inhibitors that disrupt specific Hsp90-co-chaperone interactions, or to develop Hsp90 isoform- and cell location-specific inhibitors are underway [32]. This approach of designing more precisely targeted Hsp90 inhibitors will be important to reduce undesirable pro-proliferative and mutagenic effects of Hsp90 inhibition.

## Materials and Methods

### Fly stocks and genetics

All crosses were performed at 25°C.

For the screen, *w; FRT40A*, *w; FRT42D*, *w; FRT80B*, and *w; FRT82B* strains were first isogenized for the corresponding chromosome. Males were then mutagenized with 15–25 mM elthyl methanesulfonate (EMS) and crossed to *y w ey-FLP; FRT40A* (or *42D* or *80B* or *82B*) tester virgin females which also carried the *PCNA-miniwhite^+^* reporter. Males were removed from the crosses after four days and discarded. F_1_ progeny were screened for patches of darker red eye tissue. Such flies were first re-tested by crossing again to the tester stock. If the ectopic *PCNA-miniwhite^+^* phenotype was observed in the progeny of a mutant, a stock was then established by crossing to either *y w ey-FLP GMR-lacZ; FRT42D l(2)cl-R11^1^/CyO, y^+^* (for 2^nd^ chromosome stocks) or *y w ey-FLP GMR-lacZ; FRT80B RpS17^4^/TM6B, y^+^* (for 3^rd^ chromosome stocks).

For complementation tests with known *Hsp83* alleles, *y w ey-FLP GMR-lacZ; FRT80B l(3)CCE^6-55^/TM6B, y^+^* animals were crossed to balanced stocks of known lethal alleles of *Hsp83*. If non-balancer progeny were not observed, the allelic combination was scored as lethal. Allelic combinations that produced very few non-balancer progeny (1–2 per 50 offspring) were scored as semilethal. For crosses that produced viable non-balancer progeny, male sterility was tested by individually crossing 10 non-balancer male progeny from each cross to virgin females. If none of these crosses produced progeny the allelic combinations were scored as male sterile.

Fly stocks used:

*w; FRT40A*

*w; FRT42D*

*w; FRT80B*

*w; FRT82B*

*y w ey-FLP GMR-lacZ; FRT40A; PCNA-miniwhite^+^*

*y w ey-FLP GMR-lacZ; FRT42D; PCNA-miniwhite^+^*

*y w ey-FLP GMR-lacZ; PCNA-miniwhite^+^; FRT80B*

*y w ey-FLP GMR-lacZ; PCNA-miniwhite^+^; FRT82B*

*y w ey-FLP GMR-lacZ; FRT42D l(2)cl-R11^1^/CyO, y^+^*

*y w ey-FLP GMR-lacZ; FRT80B RpS17^4^/TM6B, y^+^*

*y w ey-FLP GMR-lacZ; FRT80B l(3)CCE^6-55^/TM6B, y^+^*

*w; FRT80B ago^1^/TM6B*

*w; FRT80B ago^4^/TM6B*

*cdc2^E1-23^/CyO*

*cdc2^E1-24^/CyO*

*w; cdc2^B47^/CyO*

*w; cdc2^c03495^/CyO*

*w; cdc2^GT-000294^/CyO*

*y w; CG12082^EY20760^/TM3*

*y w ey-FLP GMR-lacZ; FRT80B*

*y w; FRT80B hs-piM(75C)*

*PCNA-GFP; FRT80B hs-piM(75C)/TM6B*

*PCNA-ΔsiteI&II-GFP; FRT80B hs-piM(75C)/TM6B*

*y w hs-FLP tub-GAL4 UAS-GFP; FRT80B tub-GAL80/TM6B*

*w; UAS-P35/CyO, act-GFP; FRT80B l(3)CCE^6-55^/TM6B*

*w; UAS-P35/CyO, act-GFP; FRT80B*

*w; Hsp83^j5C2^/TM2*

*w; Hsp83^P582^/TM6B*

*w; Hsp83^19F2^/TM3, Raf^torY9^*

*w; Hsp83^13F3^/TM6B*

*w; Hsp83^e6A^/TM6B*

*w; Hsp83^e6D^/TM6B*

*w; Hsp83^e1D^/TM6B*

*w; Hsp83^e3A^/TM6B*

*y w; Hsp83^e4A^/TM3, ftz-lacZ*

*GMR-GAL4/CyO*

*ap-GAL4 UAS-GFP/CyO*

*y v; UAS-w RNAi* (TRiP line JF01786)

*w; UAS-Hsp83 RNAi* (VDRC line 108568)

*w; UAS-Hsp83 RNAi* (NIG line 1242R-2)

*y w hs-FLP; FRT80B l(3)CCE^6-55^/TM6B*

*y w hs-FLP; P{W82}; FRT80B l(3)CCE^6-55^/SM6-TM6B* (*P{W82}* is a 7.5kb genomic rescue construct containing the *Hsp83* gene)

*w; UAS-CycE/CyO, act-GFP; FRT80B/TM6B*

*w; FRT80B Ub-GFP(13A)/TM3*

*w; CycB-GFP trap/CyO, act-GFP; FRT80B/TM6B* (*CycB-GFP trap* is line CC01846 from the FlyTrap GFP Protein Trap Database)

*w; CycB-GFP trap/CyO, act-GFP; FRT80B l(3)CCE^6-55^/TM6B*

*y w hs-FLP; FRT80B hs-piM(75C)/TM6B*

*w^1118^ UAS-Dcr-2; UAS-P35/CyO act-GFP; act>CD2>GAL4/TM6B*

*y w hs-FLP*

*y w hs-FLP; UAS-Hsp83 RNAi (NIG line 1242R-1)/TM6B*

*y w hs-FLP; Df(2L)ED6569/CyO act-GFP; UAS-Hsp83 RNAi (NIG 1242R-1)/TM6B*

*y w hs-FLP; Rca1^2^/CyO act-GFP; UAS-Hsp83 RNAi (NIG 1242R-1)/TM6B*

*y w hs-FLP; Rca1^1X^/CyO act-GFP; UAS-Hsp83 RNAi (NIG 1242R-1)/TM6B*

### Generation of *PCNA-miniwhite^+^* transgenic flies

The *pcna* promoter was amplified from genomic DNA using primers CCCAAGCTTTCCAAACCAGTTGGCAGGCCGC and CATGAATTCTGTGTTTTATTATTTAAATACTGATGACG. This PCR product was digested with HindIII and EcoRI and cloned into pBlueScript II HindIII/EcoRI sites, creating the vector pBS-PCNA. The *mini-white* gene was amplified from pUAST using primers CCGGAATTCATGGGCCAAGAGGATCAGGAG and AACTGCAGCCGAATTAATTCTAGTTCCAG. This PCR product was digested with EcoRI and PstI and cloned into pBlueScript II EcoRI/PstI sites, creating pBS-miniwhite^+^. The EcoRI/PstI fragment from pBS-miniwhite^+^ containing the *mini-white* sequence was then cloned into the EcoRI/PstI sites of pBS-PCNA, resulting in pBS-PCNA-miniwhite^+^. Finally, a SmaI/XhoI fragment from pBS-PCNA-miniwhite^+^ containing the *PCNA-miniwhite^+^* sequence was cloned into the piggyBac vector pBSII-ITR1.1k-ECFP to generate pBac.ECFP.PCNA-miniwhite^+^. Transformants were made by coinjecting embryos with pBac.ECFP.PCNA-miniwhite^+^ and pBSII-Act5c-orf, which expresses piggyBac transposase.

### Immunohistochemistry and microscopy

Pupae, staged from white prepupae (0 hr APF) at 25°C, or imaginal discs from wandering third instar larvae were dissected and fixed with 4% paraformaldehyde in PBS for 30 min at room temperature. They were then washed in PBS+0.1% Triton X-100 at least two times 10 min each. The pupal cuticle was removed from wings after fixation. Larval tissues or 24–36 hr APF pupal tissues were blocked in PBS+0.1% Triton X-100+1% BSA (PAT) for 1 hr. Pupal tissues 36–44 hr APF were blocked in PBS+0.3% Triton X-100+1% BSA overnight. All samples were incubated with primary antibody overnight at 4°C, washed with PBS+0.3% Triton X-100+0.1% BSA+2% NGS three times 20 min each, and incubated with secondary antibody conjugated to Alexa Fluor 488, 568 or 633 (Invitrogen) 1∶1000–2500 for 4 hr at room temperature. 1 µg/ml Hoechst 33258 (Invitrogen) or 0.5 µg/ml 4′,6-Diamidino-2-phenylindole (DAPI) (Sigma) were used to label nuclei and Vectashield (Vector Laboratories) was used as mounting medium.

For BrdU labeling, pupae were dissected at room temperature in Ringer's solution (Tübingen and Düsseldorf). Tissues were incubated in 100 µg/ml BrdU in Ringer's for 1 hr and then washed briefly in PBS. Fixation and labeling with α-GFP antibody was performed as usual. Following this, samples were fixed again with 100% methanol for 6 min, hydrolyzed in 2N HCl+0.1% Triton X-100 for 30 min, neutralized two times 5 min in 100 mM Borax, and washed at least three times with PBS+0.1% Triton X-100. The samples were then blocked and labeled with α-BrdU antibody per usual protocol. EdU labeling was performed similarly to the procedure for BrdU labeling, incubating for 1 hr with 10 µM EdU and using the Click-It EdU Alexa Fluor 555 imaging kit (Invitrogen) to visualize incorporation.

Wing hinge and notum, as well as head capsule and antenna, were excluded from our analyses. Confocal images were collected either on a Zeiss LSM 510 microscope (Figures 1F–F″, 2E–E′, 3B–G″, 4A–D′, 5A–C′, E–E′, G–G′, 6A–B′, F–G′, S1) or a Leica TCS SP5 microscope (Figures 2A–D′, 5D–D′, F–F′, 6C–E′, H–I′, S2) and prepared using Fiji and Adobe Photoshop CS3. All confocal images are single plane sections unless stated otherwise.

Antibodies used were: rabbit α-GFP (1∶1000; Invitrogen), mouse α-Myc 9E10 (1∶100; Santa Cruz Biotechnology), mouse α-BrdU (1∶100; BD Biosciences), mouse α-phospho-Ser10-histone H3 (PH3; 1∶1000; Millipore), rabbit α-Geminin (1∶100; provided by B. Calvi), mouse α-MPM2 (1∶200; Millipore), rabbit α-phospho-histone H1 (1∶100; Millipore), rabbit α-CycA (1∶500; provided by D. Glover), rat α-ELAV (1∶200; Developmental Studies Hybridoma Bank).

### Flow cytometry

All clones were induced at 24–48 hr after egg deposition (AED) by a 45 min heat shock in a 37°C water bath. Discs were dissected from wandering third instar larvae or from staged pupae. Dissociation of cells and FACS were performed as previously described [72]. All experiments were carried out at least three times and representative examples are shown.

### Clonal analysis

Loss-of-function clones in Figure 1, Figure 2E–E′, Figure 5E–G′ and Figure S1 were generated using mitotic recombination [73] and flippase expressed from the *eyeless* promoter (*ey-FLP*). Clones in Figure 6C–E′ and H–H′ were induced by mitotic recombination using a heat shock-inducible flippase (*hs-FLP*). All other clones were generated using MARCM [38]. MARCM mutant clones in Figure 2A–D′, Figure 4, Figure 5C–D′, Figure 6A–B′, F–G′, I–I′ and Figure S2 also overexpress P35 to inhibit apoptosis. For MARCM or *hs-FLP* experiments, larvae were heat shocked at 24–48 hr AED for 45 min. in a 37°C water bath. Larvae were collected as wandering third instars for dissection; pupae were collected for staging a 0 hr APF, aged at 25°C, and dissected at the indicated times. For experiments where mutant clones were marked by the absence of pi-Myc, pupae were heat shocked to induce expression of pi-Myc in a 37°C water bath for 30 min., beginning 90 min. prior to dissection.

**Figure 7:**
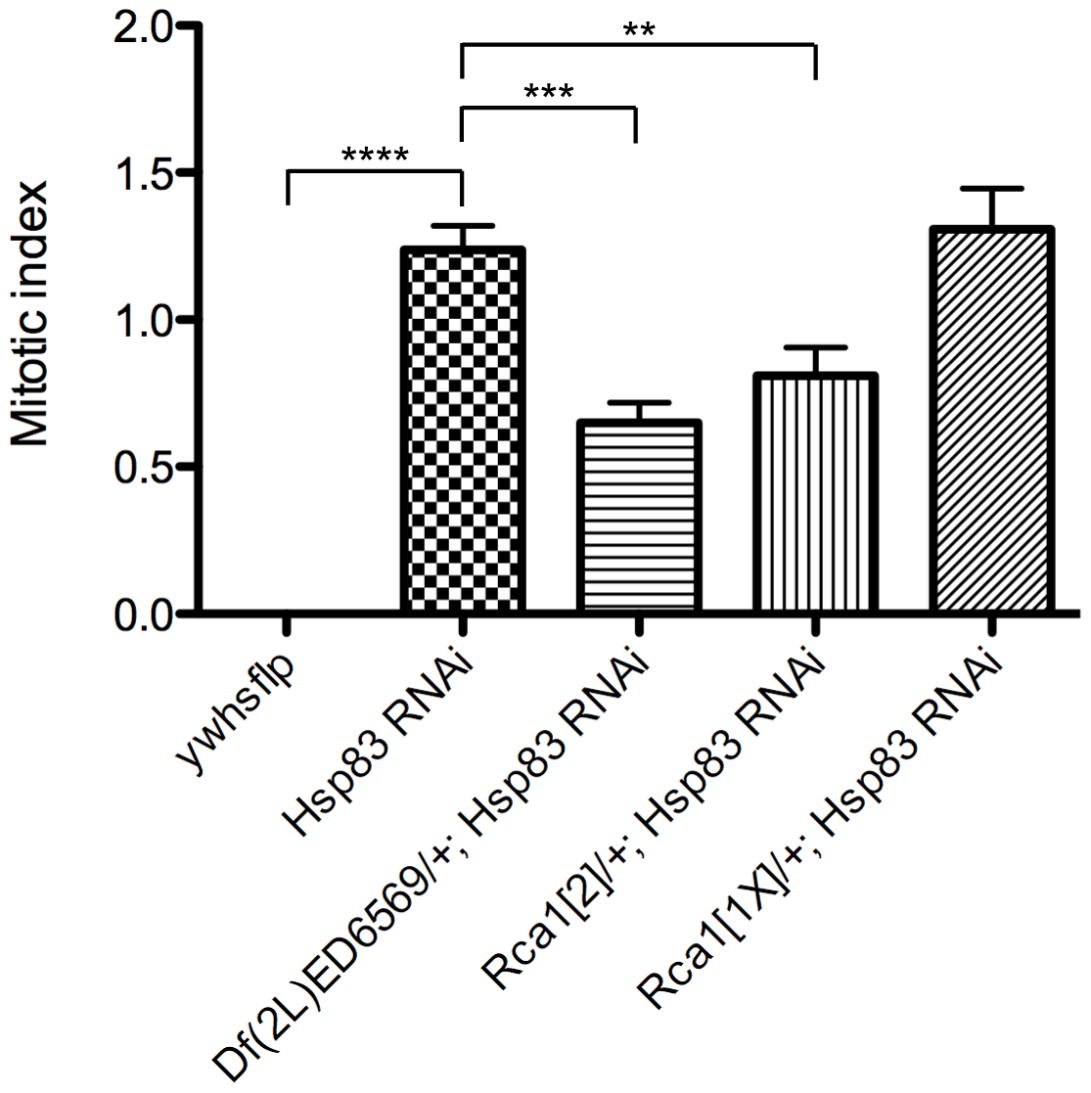
Removing one copy of *Rca1* suppresses the *Hsp83* RNAi cell cycle exit phenotype. *hs-FLP* was used to induce flip-out clones expressing *Hsp83* RNAi, Dcr-2, P35 and GFP in 28 hr APF pupal wings. These wings were then labeled with anti-PH3 and the mitotic index (the percentage of PH3-positive/GFP-positive cells) was calculated per wing. The *ywhsflp* control sample contained clones that did not express *Hsp83* RNAi. *Df(2L)ED6569* is a deletion that removes approximately 212 kb, including the entire *Rca1* coding region. 10 wings were scored for *ywhsflp*, 36 for *Hsp83* RNAi, 10 for *Df(2L)ED6569*, 18 for *Rca1^2^* and 20 for *Rca1^1X^*; a minimum of 10,000 GFP-positive cells was scored for each genotype. A Wilcoxon signed-rank test was used to determine significance between the *ywhsflp* control and the *Hsp83* RNAi samples and unpaired t tests were used to determine significance in all other cases. Bars indicate mean with SEM; ****p<0.0001, ***p = 0.0008, **p = 0.0027.

For the genetic suppression experiment (Figure 7), *w^1118^ UAS-Dcr-2; UAS-P35/CyO act-GFP; act>CD2>GAL4/TM6B* virgins were crossed to the following males:

*y w hs-FLP*

*y w hs-FLP; UAS-Hsp83 RNAi (NIG line 1242R-1)/TM6B*

*y w hs-FLP; Df(2L)ED6569/CyO act-GFP; UAS-Hsp83 RNAi (NIG 1242R-1)/TM6B*

*y w hs-FLP; Rca1^2^/CyO act-GFP; UAS-Hsp83 RNAi (NIG 1242R-1)/TM6B*

*y w hs-FLP; Rca1^1X^/CyO act-GFP; UAS-Hsp83 RNAi (NIG 1242R-1)/TM6B*

Progeny from each cross were heat shocked in a 37°C water bath for 8 min. at 96 hr AED to induce clones. Non-act-GFP Tb+ female 0 hr APF pupae were collected, aged at 25°C until 28 hr APF and dissected and labeled with anti-PH3 antibodies. The percentage of PH3-positive cells per GFP+ clone cell per wing was scored blind, and a minimum of 10 wings and 10,000 GFP+ cells were counted for each genotype. Unpaired t tests and Wilcoxon signed-rank test were performed with GraphPad Prism software.

### Mapping of the *l(3)CCE^6-55^* mutant

Initial mapping of the gene corresponding to *l(3)CCE^6-55^* was performed by crossing the *l(3)CCE^6-55^* stock to the Bloomington 3L Deficiency kit. Genetic complementation was determined by scoring non-balancer progeny for lethality. *l(3)CCE^6-55^* failed to complement *Df(3L)BSC23*, which contains a deletion removing cytological region 62E8-63B6. We then molecularly mapped the deletion in *Df(3L)BSC23* by performing inverse PCR for a lacW element on *Df(3L)BSC23* flies, as *Df(3L)BSC23* was originally isolated as a P transposase-induced male recombination event between two lacW P elements. This analysis indicated the *Df(3L)BSC23* deletion removes everything between genomic coordinates 2590407–3193245 (∼600 kb). We confirmed this by performing PCR across the deletion breakpoint and sequencing the PCR product.

Additional complementation crosses to smaller molecularly defined deficiency lines within this region revealed that *l(3)CCE^6-55^* complemented *Df(3L)BSC119*, *Df(3L)Exel6091*, *Df(3L)Exel6092*, and *Df(3L)ED4288*. The genomic regions deleted in these lines therefore did not contain the *6-55* mutation. This left three regions remaining that could contain the gene corresponding to *6-55*: 2590407–2600282, 3047162–3070827, and 3149091–3193245.

In parallel to the deficiency mapping, recombination mapping of the *l(3)CCE^6-55^* lethal mutation relative to molecularly defined transposable element insertions was also performed [74]. Two rounds of mapping (rough and fine) indicated that the *6-55* mutation resided extremely close to P insertion *P{SUPor-P}CG14965^KG05766^* at 3L coordinate 3192433, which is at the very proximal end of the deletion in *Df(3L)BSC23*. This indicated that the *l(3)CCE^6-55^* mutation likely resided in the ∼50 kb region 3149091–3193245 defined on the left by the proximal breakpoint of *Df(3L)ED4288* and on the right by the proximal breakpoint of *Df(3L)BSC23*. The endpoints of the *Df(3L)ED4288* deletion were also confirmed by PCR and sequencing.

The distal end of this ∼50 kb region of interest (ROI) was within the *BTBVII* gene, and the proximal end lay within the *Hsp83* gene. We therefore designated the entire region between the next distal neighboring gene to *BTBVII* and the next proximal neighboring gene to *Hsp83* as the region to be sequenced in both *l(3)CCE^6-55^* and the isogenic parental strain to identify mutations.

For sequencing the ∼50 kb ROI, DNA was isolated from homozygous *w; FRT80B* and *y w ey-FLP GMR-lacZ; FRT80B l(3)CCE^6-55^* animals. High fidelity Pfx50 DNA polymerase (Invitrogen) was used to amplify 2–5 kb portions of the ROI from genomic DNA of both genotypes. These PCR products were sequenced at the Fred Hutchinson Cancer Research Center Genomics core facility and sequence reads for *FRT80B* and *l(3)CCE^6-55^* were aligned using DNA Strider software. A total of 39,53 bp of the ROI was sequenced in both genotypes and they were identical except for a single nucleotide change at 3L coordinate 3195636, in the *Hsp83* gene.

All genomic coordinates given are based on the *Drosophila* genome version 5.8, released May 30, 2008.

## Supporting Information

Figure S1 *PCNA-GFP* reporter activation in *6-55* mutant clones is dependent on the E2F binding sites. (A) *6-55* mutant mitotic clones marked by the absence of pi-Myc marker (red) in a pupal eye after 24 hr APF. (B) A mutant *PCNA-GFP* reporter lacking the E2F binding sites in the *pcna* enhancer/promoter sequence is not expressed in *6-55* mutant clones. (C) Merged image. Yellow arrows indicate the positions of two mutant clones.
(TIF)

Figure S2 *6-55* mutant cells ectopically proliferate until approximately 40 hr APF. The MARCM system was used to induce clones overexpressing GFP and the P35 apoptosis inhibitor and homozygous for either a wild-type FRT80B chromosome (A–B′, E–F′) or an FRT80B chromosome containing the *6-55* mutation (C–D′, G–H′). Pupal eyes (A–D′) and wings (E–H′) were isolated at 39–41 hr APF and assayed for EdU incorporation (A–A′, C–C′, E–E′, G–G′; red) or PH3 staining (B–B′, D–D′, F–F′, H–H′; red). Clones are marked by the presence of GFP (green) and DNA was stained with DAPI (blue). White arrows indicate the presence of proliferation markers in *6-55* mutant clones. All scale bars are 25 µm.
(TIF)

Table S1 Results of the *PCNA-miniwhite+* EMS screen. Summary of the loss-of-function screen results per chromosome arm. The number of F1 progeny screened, number of stocks established that exhibited *PCNA-miniwhite+* expression, and the number and identity of mutant lines with a confirmed cell cycle exit delay are indicated.
(DOC)
