# Supplementary figures and images for: The Molecular Chaperone Hsp90 Is Required for Cell Cycle Exit in Drosophila melanogaster
Source: PLoS Genet. 2013 Sep 26;9(9):e1003835. doi: 10.1371/journal.pgen.1003835 (PMC3784567; doi:10.1371/journal.pgen.1003835)

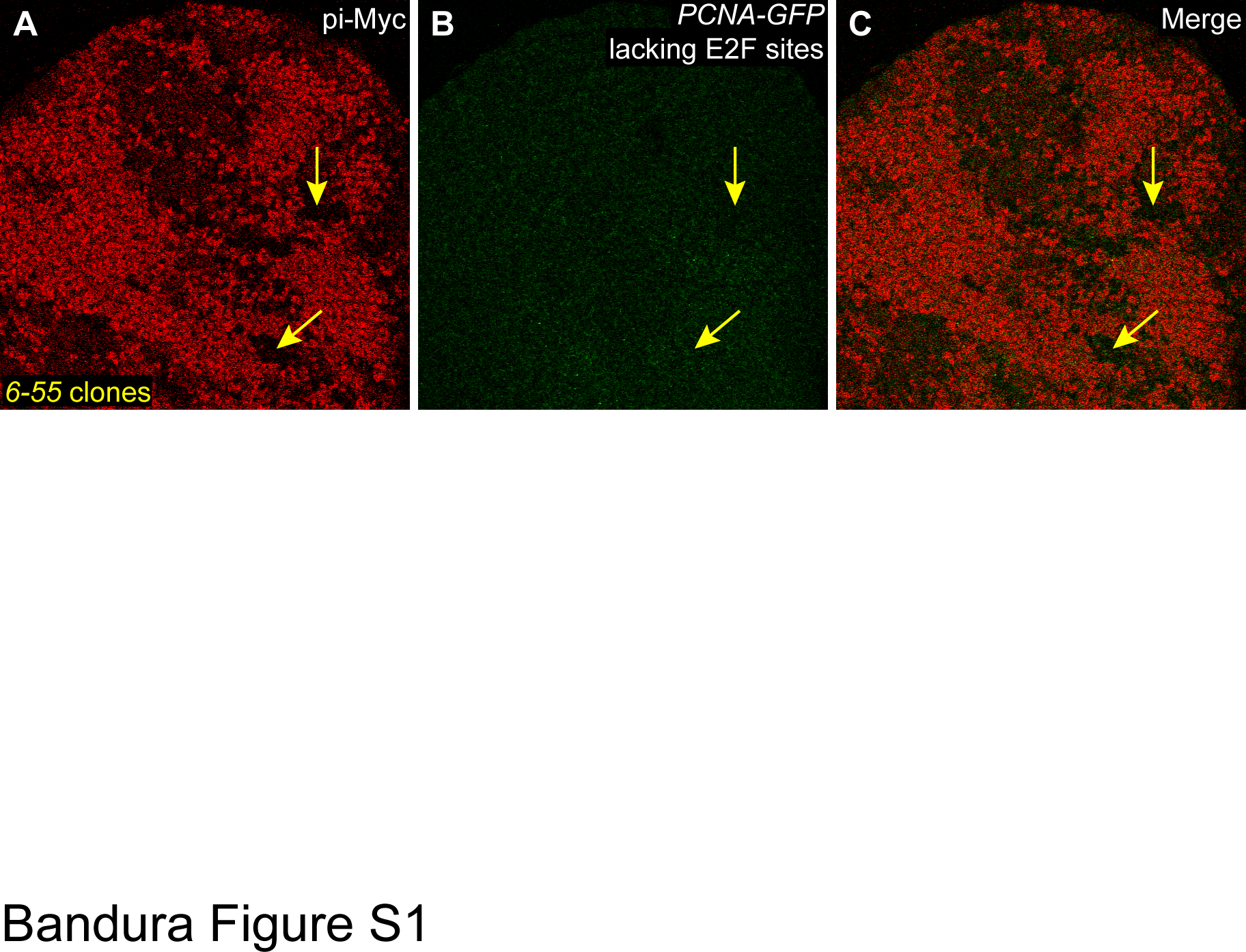

Supplement: Figure S1 — PCNA-GFP reporter activation in 6-55 mutant clones is dependent on the E2F binding sites. (A) 6-55 mutant mitotic clones marked by the absence of pi-Myc marker (red) in a pupal eye after 24 hr APF. (B) A mutant PCNA-GFP reporter lacking the E2F binding sites in the pcna enhancer/promoter sequence is not expressed in 6-55 mutant clones. (C) Merged image. Yellow arrows indicate the positions of two mutant clones. (TIF) [file pgen.1003835.s001.tif]

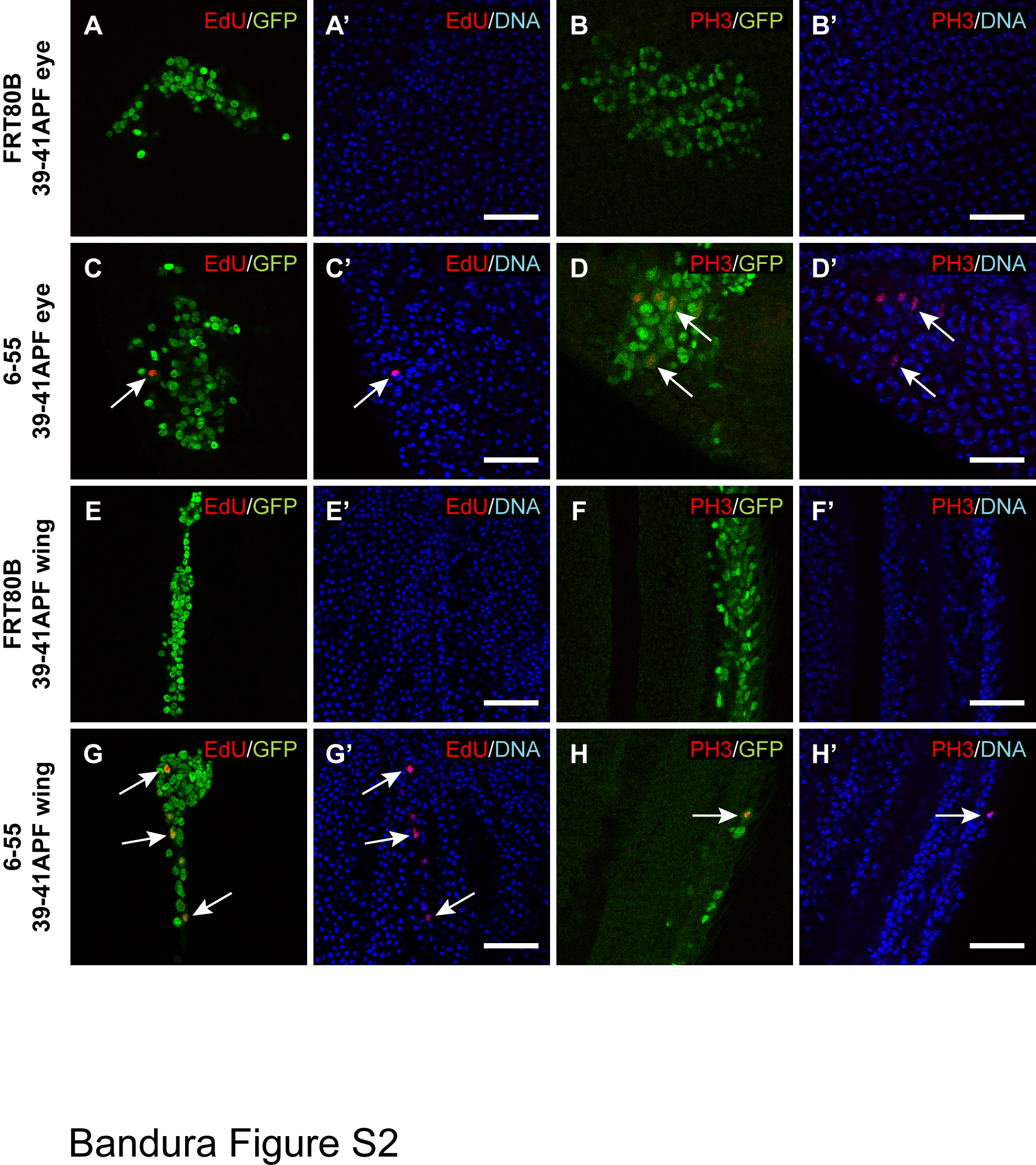

Supplement: Figure S2 — 6-55 mutant cells ectopically proliferate until approximately 40 hr APF. The MARCM system was used to induce clones overexpressing GFP and the P35 apoptosis inhibitor and homozygous for either a wild-type FRT80B chromosome (A–B′, E–F′) or an FRT80B chromosome containing the 6-55 mutation (C–D′, G–H′). Pupal eyes (A–D′) and wings (E–H′) were isolated at 39–41 hr APF and assayed for EdU incorporation (A–A′, C–C′, E–E′, G–G′; red) or PH3 staining (B–B′, D–D′, F–F′, H–H′; red). Clones are marked by the presence of GFP (green) and DNA was stained with DAPI (blue). White arrows indicate the presence of proliferation markers in 6-55 mutant clones. All scale bars are 25 µm. (TIF) [file pgen.1003835.s002.tif]
