# Supplementary material for: The Molecular Chaperone Hsp90 Is Required for Cell Cycle Exit in Drosophila melanogaster
Source: PLoS Genet. 2013 Sep 26;9(9):e1003835. doi: 10.1371/journal.pgen.1003835 (PMC3784567; doi:10.1371/journal.pgen.1003835)
Supplement: Table S1 — Results of the PCNA-miniwhite+ EMS screen. Summary of the loss-of-function screen results per chromosome arm. The number of F1 progeny screened, number of stocks established that exhibited PCNA-miniwhite+ expression, and the number and identity of mutant lines with a confirmed cell cycle exit delay are indicated. (DOC) [file pgen.1003835.s003.doc]

**Table S1. Results of the *PCNA-miniwhite+* EMS screen.**

|  | Chromosome arm | | | |  |
| --- | --- | --- | --- | --- | --- |
|  | 2L | 2R | 3L | 3R | Total |
| F1 progeny screened^a^ | ~110,000 | ~7,000 | ~66,000 | ~2,500 | ~185,500 |
| *PCNA-miniwhite+* stocks established^b^ | 23 | 5 | 27 | 6 | 61 |
| *PCNA-GFP+*^c^ | 2(1) | 0 | 8(5) | 0 | 10(6) |
| Gives ectopic cell divisions^d^ | 2(1) | N/A | 6(4) | N/A | 8(5) |
| Complementation groups and alleles (and genes, if mapped) | *l(2)CCE3^#28^*  *l(2)CCE3^#30^* (*cdc2*) | N/A | *l(3)CCE6^1-55^* *l(3)CCE6^4-50^* *l(3)CCE6^4-61^* (*ago*) | N/A |  |
|  |  |  | *l(3)CCE2^6-55^* (*Hsp83*) |  |  |
|  |  |  | *l(3)CCE4^5-94^* (*CG12082*) |  |  |
|  |  |  | *l(3)CCE5^4-95^* |  |  |

^a^ The flies screened were the F1 progeny of a cross between mutagenized males carrying an FRT chromosome and virgin females expressing *ey-FLP*, the *PCNA-miniwhite+* reporter, and containing an appropriate wild-type FRT chromosome.

^b^ The number of mutant stocks established for each chromosome arm that resulted in increased *PCNA-miniwhite+* expression.

^c^ The number of mutant lines found to also cause increased expression of a *PCNA-GFP* reporter in pupal eyes after 24 hours APF. The number in parentheses represents the number of complementation groups these lines fall into.

^d^ Determined by the presence of BrdU-positive and PH3-positive cells in mutant clones in pupal eyes and wings after 24 hours APF.
